# Genome-wide differential expression profiling of lncRNAs and mRNAs in human induced pluripotent stem cell-derived endothelial cells exposed to e-cigarette extract

**DOI:** 10.1186/s13287-021-02654-6

**Published:** 2021-12-04

**Authors:** Hoai Huong Thi Le, Chen-wei Liu, Philip Denaro, Jordan Jousma, Ning-Yi Shao, Irfan Rahman, Won Hee Lee

**Affiliations:** 1 Department of Basic Medical Sciences, University of Arizona College of Medicine, 425 N 5th Street, Building ABC1, Rm 426, Phoenix, AZ 85004-2157 USA; 2 Department of Pharmacology and Regenerative Medicine, University of Illinois College of Medicine, Chicago, IL 60612 USA; 3 Health Sciences, University of Macau, Macau, China; 4 Department of Environmental Medicine, University of Rochester Medical Center, Rochester, NY 14642 USA

**Keywords:** lncRNAs, e-cigarettes, iPSC-ECs, fatty acid oxidation, endothelial dysfunction

## Abstract

**Background:**

Electronic-cigarette (e-cig) usage, particularly in the youth population, is a growing concern. It is known that e-cig causes endothelial dysfunction, which is a risk factor for the development of cardiovascular diseases; however, the mechanisms involved remain unclear. We hypothesized that long noncoding RNAs (lncRNAs) may play a role in e-cig-induced endothelial dysfunction.

**Methods:**

Here, we identified lncRNAs that are dysregulated in human induced pluripotent stem cell-derived endothelial cells (iPSC-ECs) following 24 h of e-cig aerosol extract treatment via microarray analysis. We performed Gene Ontology and Kyoto Encyclopedia of Genes and Genome (KEGG) pathway analyses of the dysregulated mRNAs following e-cig exposure and constructed co-expression networks of the top 5 upregulated lncRNAs and the top 5 downregulated lncRNAs and the mRNAs that are correlated with them. Furthermore, the functional effects of knocking down lncRNA lung cancer-associated transcript 1 (LUCAT1) on EC phenotypes were determined as it was one of the significantly upregulated lncRNAs following e-cig exposure based on our profiling.

**Results:**

183 lncRNAs and 132 mRNAs were found to be upregulated, whereas 297 lncRNAs and 413 mRNAs were found to be downregulated after e-cig exposure. We also observed that e-cig caused dysregulation of endothelial metabolism resulting in increased FAO activity, higher mitochondrial membrane potential, and decreased glucose uptake and glycolysis. These results suggest that e-cig alters EC metabolism by increasing FAO to compensate for energy deficiency in ECs. Finally, the knockdown of LUCAT1 prevented e-cig-induced EC dysfunction by maintaining  vascular barrier, reducing reactive oxygen species level, and increasing migration capacity.

**Conclusion:**

This study identifies an expression profile of differentially expressed lncRNAs and several potential regulators and pathways in ECs exposed to e-cig, which provide insights into the regulation of lncRNAs and mRNAs and the role of lncRNA and mRNA networks in ECs associated e-cig exposure.

**Supplementary Information:**

The online version contains supplementary material available at 10.1186/s13287-021-02654-6.

## Introduction

Electronic-cigarette (e-cig), a form of electronic nicotine delivering system (ENDS), functions by the vaporization of a solution, termed e-liquid, containing propylene glycol (PG) and/or vegetable glycerin (VG), flavoring agents, and with or without nicotine. The general consensus, when comparing e-cigs to traditional cigarettes, is that e-cigs are less toxic [1–7]. Perhaps, due to marketing centered around the relative safety of e-cigs, their popularity in recent years has challenged the market share of traditional combustible nicotine cigarettes. More alarmingly, e-cigs have found an audience among adolescents, causing the US Surgeon General to declare ENDS usage in youths an epidemic [8]. Subsequently, the FDA issued a statement to limit the sale of e-liquid flavors, excluding tobacco, mint, and menthol to curb the popularity of e-cigs in the youth population. Regulating flavorings used in e-cig manufacturing is supported by recent studies showing that flavoring agents, particularly cinnamaldehyde, the major ingredient in cinnamon flavors, is responsible for the toxicities observed [9–11]. In addition, nicotine has been shown to be detrimental to adolescent development, and exposure to nicotine early in life increases risk of addiction [12]. To date, there are few studies looking at effects of using e-cigs beyond the respiratory system. Studies examining e-cigs in the cardiovascular context are limited, though there are studies that conclude detrimental effects on endothelial function, and their system effects [11, 13, 14].

Only a small portion of the human genome contains coding sequences. Historically, the noncoding regions were considered extraneous information without function beyond space fillers between protein-coding genes; however, to date, noncoding RNAs (ncRNAs) have been shown to have regulatory functions at both the transcriptional and posttranscriptional levels [15, 16]. Long noncoding RNAs (lncRNAs) are a class of ncRNAs with sequences of at least 200 nucleotides long. Many lncRNAs have been found to be dysregulated in smokers or cells treated with cigarette smoke extract (CSE). For example, LUCAT1, also referred to as the smoke and cancer-associated lncRNA 1 (SCAL1), was induced by cigarette smoke in primary bronchial epithelial cells and has been also shown to regulate gene expression and mediate a protective response against oxidative stress [17]. LncRNA TUG1 was also found to be upregulated in human bronchial epithelial cells and lung fibroblasts treated with CSE [18]. To date, the effects of e-cigs on lncRNAs have not been characterized thoroughly.

Endothelial cells (ECs) predominately utilize glucose as an energy source; however, certain conditions can cause ECs to metabolize fatty acids. Kalucka et al. demonstrated that ECs upregulate fatty acid oxidation (FAO) during quiescence to regulate homeostasis [19]. There is no consensus in the metabolic changes following exposure to cigarettes, and minimal studies have examined this effect in the context of e-cigs. Following CSE exposure, primary mouse pulmonary microvascular ECs reduced mitochondrial respiration and FAO [20]. Conversely, bronchial epithelial cells and alveolar cells exposed to CSE and cigarette smoke, respectively, had an increase in FAO [21, 22]. In our previous study, acute exposure to flavored e-liquids or serum from e-cig users caused endothelial dysfunction [11]. Here, we first treated human induced pluripotent stem cell-derived endothelial cells (iPSC-ECs) from four healthy donors with menthol-flavored e-cig aerosol extract (EAE), the most common flavor for young adults [23], and observed endothelial dysfunction. Microarray was then used to identify lncRNAs and mRNAs that are differentially expressed in iPSC-ECs after exposure to EAE. Through bioinformatics, we examined pathways that may be altered following e-cig exposure and built an mRNA–lncRNA co-expression network. We also examined the changes in FAO and glycolysis after exposure of EAE. Further, we investigated the functional effects of knocking down lncRNA LUCAT1, which was significantly upregulated after EAE treatment, on EC phenotypes in order to characterize its role in e-cig-induced endothelial dysfunction.

## Materials and methods

### Differentiation of iPSC-ECs

iPSCs with passage numbers over 20 from four healthy biological donors were split at a ratio 1:12 using EDTA and grown until they reached approximately 75% confluency. To initiate differentiation into endothelial cell [24], iPSCs were treated with N2B27 medium supplemented with 6 μM CHIR + 25 ng/ml BMP4 on day 0. For days 3–5, the medium was replaced with StemPro-34 medium supplemented with 100 ng/ml VEGF and 2 μM forskolin. On day 6, endothelial cells were isolated using CD144 MicroBeads and magnetic cell sorting (MACS) system (Miltenyi Biotech), according to the manufacturer’s protocol, and expanded on fibronectin-coated plates. iPSC-ECs were cultured in Endothelial Growth Medium-2 (EGM2, Lonza Bioscience) medium at 37ºC and 5% CO_2_ with medium changes every 48 h. EC differentiation protocol is shown in Additional file 1: Fig. S1A. Experiments described in this manuscript were performed between passages 1 and 4.

### Characterization of iPSC-ECs

Endothelial differentiation efficiency and EC phenotypes were assessed and confirmed by performing flow cytometry analysis, low-density lipoprotein (LDL) uptake assay, and immunofluorescence staining (Additional file 1: Figs. S1B and S1C). For flow cytometry, the cells were directly labeled with PE-conjugated CD31 antibody (Cat#: 555446; BD Biosciences), FITC-conjugated CD144 antibody (Cat#: 560411; BD Biosciences), APC-eFluor780-conjugated CD45 (Cat#: 50-245-972; Thermo Fisher Scientific), and FITC-conjugated CD34 antibody (Cat#: 560942; BD Biosciences) for 15 min in the dark at 4 °C and analyzed using a LSR II flow cytometer. Flow cytometry was performed at the UA College of Medicine—Phoenix Core Facility. LDL uptake was assessed by incubating cells with Dil Ac-LDL (L3484, Thermo Fisher Scientific) in EGM2 medium for 4 h at 37 °C. Cells were then fixed with 4% paraformaldehyde (PFA) for 10 min and mounted with mounting medium containing DAPI, and fluorescence microscopy was used to capture images. For immunostaining, the cells were fixed in 4% PFA, permeabilized in 0.1% Triton-X, and blocked with 10% donkey serum in PBS for an hour. The cells were then incubated with VE-cadherin (VE-CAD) antibody (AF938; R&D systems) in blocking buffer overnight at 4 °C. After washing with PBS, cells were incubated with Alexa Fluor-488 donkey anti-goat IgG (A32814, Thermo Fisher Scientific). Cells were then mounted with mounting medium containing DAPI, and fluorescence microscopy was used to capture images.

### EAE preparation

E-cig aerosol was produced with a Vision Spinner II attached to a Kanger Mini Protank 3 housing a Kanger V2 1.8 Ohm coil using a previously described smoking machine [4, 25, 26]. The glassomizer was filled with menthol-flavored e-liquid (50%/50% PG/VG; purchased from the Vape Dudes) with a labeled nicotine concentration of 24 mg/ml. The puffing protocol was based on Health Canada Intense puffing regime [27, 28] using the following parameters: 2-s puff duration, every 30 s, with a 55-ml puff volume. The solution was then sterile-filtered using a 0.33-μm filter. A stock solution of 15 TPE was made by puffing 60 puffs into 4 ml of EGM2 media. Nicotine concentration was determined by measuring the mass of the glassomizers before and after extractions and the density of the e-liquid as described previously to maintain consistent generation of e-cig vapors across all extraction [29].

### Cell viability, ROS production, and caspase 3/7 activity

Cells were analyzed for viability, ROS generation, and caspase 3/7 activity using PrestoBlue Cell Viability Reagent (Thermo Fisher Scientific), ROS-Glo H_2_O_2_ (Promega), and Caspase-Glo 3/7 (Promega), respectively, per manufacturer’s protocol. Briefly, cells were plated in a 96-well plate at a density of 4.0 × 10^3^ cells/well and treated with varying dilutions of EAE (0–10 TPE) when cells reached 60% confluency. Cell viability was assessed using 10% PrestoBlue Cell Viability Reagent diluted in EGM2 medium. ROS levels were measured after cells were incubated with diluted EAE for 48 h. Six hours prior to the completion of treatment, ROS substrate was added to the wells. Fifty microliters of media from the samples was transferred to a 96-well white plate with 50 μl of detection solution added. Following 30-min incubation, luminescence signal was measured using a plate reader. In addition, the activity of caspase 3/7 was assessed by adding 100 μl Caspase-Glo 3/7 reagent into each well for an hour and total luminescence was measured. Calcein AM (Thermo Fisher Scientific) was added to the original plate containing cells to measure cell number and normalize ROS production and caspase 3/7 activity.

### Measurement of endothelial function

EC tube formation was carried out following manufacturer’s suggested protocol for 15-well μ-Slide Angiogenesis (ibidi). In short, after coating each well with Matrigel Basement Membrane Matrix (Corning), 1.0 × 10^4^ cells were seeded in EAE diluted in EGM2 medium. Following 16-h incubation, capillary network images were captured and analyzed via ImageJ using the Angiogenesis Analyzer plugin.

For migration assay, cells were grown to 100% confluency in a 24-well plate and a sterile 200-µl pipette tip was used to generate a cell-free zone. Cells were then washed with DPBS before being replaced with either diluted EAE in EGM2 media or EGM2 media only. Images were captured at 0, 16, and 24 h and analyzed via ImageJ using the MRI Wound Healing Tool plugin.

For flow cytometry analysis, manufacturer’s protocol for FITC Annexin V/Dead Cell Apoptosis Kit was used and analyzed using a LSR II flow cytometer. In short, cells were harvested, washed, and stained with Annexin-V and PI in each sample for 15 min at room temperature. Flow cytometry was performed at the UA College of Medicine—Phoenix Core Facility.

For permeability analysis, cells were grown until full confluency on Transwell Permeable Supports (Corning). Cells were then treated with diluted EAE or EGM2 for 24 h. Medium from the top and bottom chambers was removed, and the bottom chamber was replaced with fresh medium. Medium containing streptavidin–HRP (Pierce) was added to the top of the inserts and incubated for 5 min at 37 °C. The inserts were removed, and 20 μl of the bottom chamber was transferred to a 96-well plate. Fifty microliters of Turbo TMB-ELISA Substrate Solution (Pierce) was added to the medium and incubated at room temperature until the reaction turns blue and stabilizes. Fifty microliters of Stop Solution for TMB Substrates (Thermo Fisher Scientific) was then added, and plates were measured for absorbance at 450 nm.

### Microarray and bioinformatics analysis

Total RNA from four biological samples was extracted using a Direct-zol RNA Kit (Zymo Research) following manufacturer’s protocol. RNA concentration, integrity, and purity were assessed using a NanoDrop ND-1000, Bioanalyzer 2100 (Agilent Technologies, USA), and denaturing gel, respectively. The sample preparation and microarray hybridization were performed according to manufacturer’s protocols.

The profiling of lncRNAs and protein-coding mRNAs was performed using Arraystar Human LncRNA Arrays V5.0 (Arraystar Inc.), which detects 39,317 lncRNAs and 21,174 protein-coding transcripts. The acquired array images scanned by the Agilent Scanner G2505C were analyzed by Agilent Feature Extraction software (version 11.0.1.1). GeneSpring GX v12.1 software package (Agilent Technologies) was used for quantile normalization and subsequent data processing; quantile-normalized lncRNAs and mRNAs with “Present” or “Marginal” flags in at least 4 out of the eight samples were further analyzed. *p* value (< 0.05)/FDR filtering (fold change ≥ 2.0) was used to identify differentially expressed lncRNAs and mRNAs. Volcano plot filtering and hierarchical clustering between untreated group and treated group were performed to show the expression profile of the differentially expressed lncRNAs and mRNAs with statistical significance. GO and KEGG pathway analyses were performed on the differentially expressed lncRNA co-expressed mRNAs (*p* < 0.05).

### LncRNA-mRNA co-expression network analysis

To reveal potential association of the differentially expressed lncRNAs with mRNAs, the PCCs were calculated on the top 5 upregulated and downregulated differentially expressed lncRNAs and all mRNAs and those pairs with significant correlations (│PCC│ ≥ 0.9, *p* value ≤ 0.05) were chosen to construct the network. In these representations, each lncRNA and gene corresponded to red and blue nodes, respectively, and solid lines between nodes denote a positive PCC, whereas dashed lines denote a negative PCC.

### Quantitative real-time reverse transcription polymerase chain reaction (RT-PCR)

To validate microarray findings, total RNA from four biological replicates was extracted using a Direct-zol RNA kit (Zymo Research) according to manufacturer’s instruction. The first-strand cDNA was synthesized from 500 ng of RNA using a RT^2^ first-strand kit (Qiagen) and high-capacity cDNA reverse transcription kit (Applied Biosystems) according to the manufacturer’s protocol. RT-PCR assay was carried out in a QuantStudio Flex 6 (Applied Biosystem, USA) using RT^2^ SYBR Green Mastermix (Qiagen), RT^2^ lncRNA PCR Array (Qiagen), and PowerUp SYBR Green Master Mix (Thermo Fisher Scientific). The expression level of each lncRNA is presented as the fold change of each lncRNA and was calculated using the 2^−ΔΔCt^ formula and normalized to the expression of GAPDH. All primers are provided in Additional file 1: Table S1.

### FAO activity assay

FAO Assay Kit (Biomedical Research Service Center, State University of New York, Buffalo, NY) was used according to manufacturer’s protocol. In short, cells treated with diluted EAE or basal medium were harvested with 1 × cell lysis solution. Fifty microliters of the FAO Assay Solution or control solution was added to 10 μl of the cell lysate. The reactions were incubated at 37 °C for 2 h before termination by the addition of 50 μl of 3% acetic acid, and the OD_492nm_ was measured using a plate reader. Control reaction reading was subtracted from the FAO reaction reading. FAO activity was normalized by protein concentration, as assessed via BCA assay, and to the activity of the control sample.

### Glycolysis assay

Glycolysis cell-based assay (Cayman Chemical) was used to measure L-lactate levels according to manufacturer’s protocol. In short, cells were treated with diluted EAE or basal medium. Following 24-h incubation, cells were spun down at 1000 rpm for 5 min and 10 μl of supernatant was transferred to a new plate containing 90 μl of assay buffer. Hundred microliters of reaction solution was added to each well, and the reactions were incubated on an orbital shaker for 30 min at room temperature. A plate reader was used to read absorbance at 490 nm and compared to L-lactate standards.

### Glucose uptake assay

Glucose uptake was measured using Glucose Uptake-Glo (Promega) according to manufacturer’s recommended protocol. In short, cells were seeded in a 96-well plate. After a 24-h treatment with EAE or basal medium, 50 μl of supernatant was removed from the microplate. Fifty microliters of the glucose detection reagent was added to each well and mixed for a minute on an orbital shaker. The luminescence was measured after an hour incubation at room temperature.

### Mitochondrial staining

Following 48-h EAE treatment, cells were stained with 100 nM MitoTracker Red FM (Invitrogen) and 100 μM MitoView Green (Biotium) for 30 min at 37 °C. Following washing with PBS, cells were placed in live-cell imaging solution for live cell imaging. Mean fluorescence intensity was quantified using ImageJ. For flow cytometry analysis, cells were incubated with MitoTracker Red 100 nM for 45 min and analyzed using a LSR II flow cytometer.

### Intracellular ATP quantification

ATP levels were measured using CellTiter-Glo 2.0 Cell Viability Assay (Promega) according to manufacturer’s protocols. In short, cells were plated in a white 96-well microplate and treated with EAE and/or 1.5 μM of oligomycin A for 48 h. Fifty microliters of supernatant was removed from the microplate. Fifty microliters of the CellTiter-Glo 2.0 Reagent was added to each well and mixed for 2 min on an orbital shaker. The luminescence was measured after a 10-min incubation at room temperature.

### Small interfering RNA transfections

Small interfering RNAs (siRNAs) targeting negative control (Thermo Fisher Scientific) and LUCAT1 (Cat# N-190814–01-0005; Horizon Discovery) were transfected when cells reached 70% confluent at a final concentration of 25 nM using Lipofectamine RNAiMax (Thermo Fisher Scientific) for 24 h prior to assays according to manufacturer’s protocols.

### Statistical analysis

Statistical analysis and graphs were performed and created with GraphPad Prism. Data were presented as the mean ± standard deviation (SD). Unpaired two-tailed Student’s t-tests for normally distributed samples and Mann–Whitney U test for non-normally distributed samples were used to measure significance, with *p* values < 0.05 considered to be statistically significant.

## Results

### Exposure to e-cig leads to EC dysfunction

**Fig. 1:**
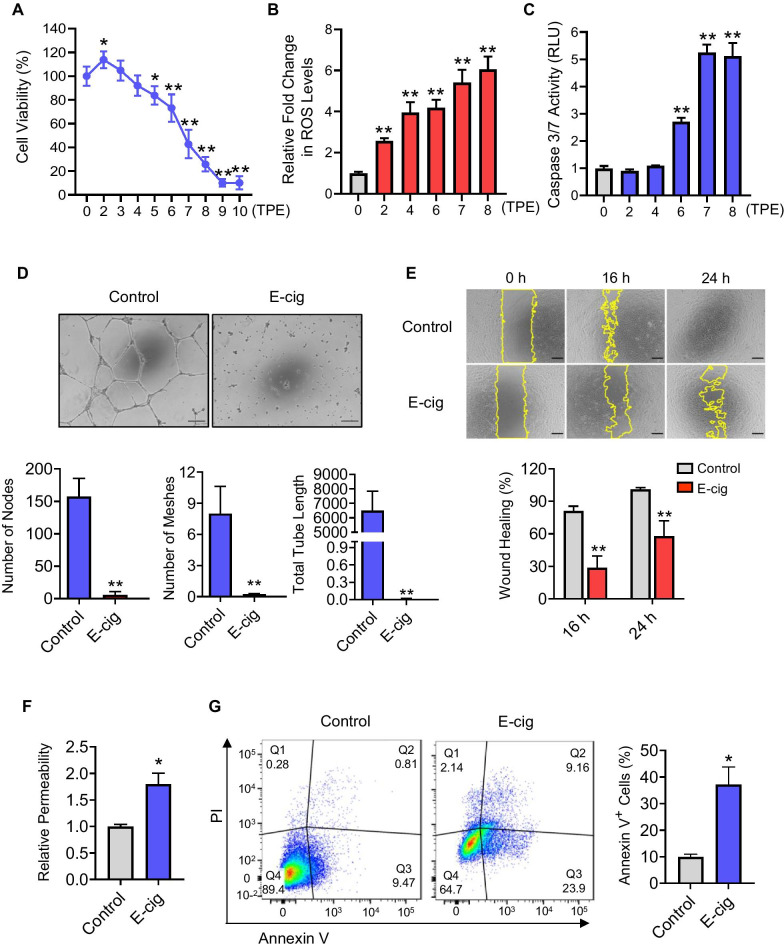
Assessment of the biological effects of e-cig on iPSC-ECs. **A** Cell viability, **B** ROS production, and **C** caspase 3/7 activity were assessed by exposing cells to various concentrations (2–10 TPE) of menthol-flavored EAE for either 24 or 48 h. **D** Tube formation ability was also examined by exposing cells to 6.5 TPE EAE for 16 h to form capillary-like structure and then imaged via phase-contrast microscopy (10 x). **E** Representative images and quantitative data analysis of migration assays preformed on cells treated with 6.5 TPE EAE at 0, 16, and 24 h. **F** Permeability of ECs was measured by the transwell permeability assay with streptavidin–HRP and TMB following exposure to 6.5 TPE EAE for 24 h. **G** Flow cytometric data for the Annexin V and PI staining of cells treated with 6.5 TPE EAE. Data were obtained using iPSC-ECs from four healthy donors, and the assays were repeated three times. Data are represented as mean ± SD. * and ** indicate *p* < 0.05 and *p* < 0.001, respectively. Scale bars = 500 μm.

To assess the effects of e-cig on EC phenotype and determine the appropriate range of EAE for transcriptomic profiling, iPSC-ECs differentiated from four healthy individuals-derived iPSCs (Additional file 1: Fig. S1) were treated with serial dilutions of menthol-flavored EAE at 24 mg/ml of nicotine for either 24 or 48 h. We found that there was a direct correlation between concentration and cell death with significantly increased cell death beginning at 5 TPE (total puff equivalent; puffs per mL of medium [30]) and almost complete cell death at 9 and 10 TPE (Fig. 1A) after 24 h EAE treatment. Upregulated reactive oxygen species (ROS) production is associated with the pathogenesis of vascular diseases [31]; accordingly, we found that e-cig enhanced ROS production in a concentration-dependent manner (Fig. 1B). In addition, consistent with our previous study [11], iPSC-ECs exposed to EAE exhibited increased apoptotic activity as indicated by significantly increased caspase 3/7 activity at 6, 7, and 8 TPE (Fig. 1C). Endothelial function was further analyzed by utilizing tube formation and migration assays. Compared to the carrier control, iPSC-ECs treated with EAE produced a significantly lower number of nodes and meshes and shorter tubes (Fig. 1D). Similarly, e-cig exposure impeded the migration ability (Fig. 1E) and barrier function (Fig. 1F) of iPSC-ECs. Moreover, flow cytometric analysis of iPSC-ECs treated with EAE resulted in significantly increased cell numbers with Annexin V-FITC and propidium iodine (PI) staining (Fig. 1G). Along with the decrease in viable cells (Q4), there was an increase in apoptotic Annexin + /PI- cells (Q3), late apoptotic Annexin + /PI + (Q2), and necrotic Annexin-/PI + cells (Q1) following EAE treatment. Taken together, endothelial phenotypes and function are significantly impaired by e-cig treatment.

### Identification of differentially expressed lncRNAs and mRNAs following e-cig exposure

**Fig. 2:**
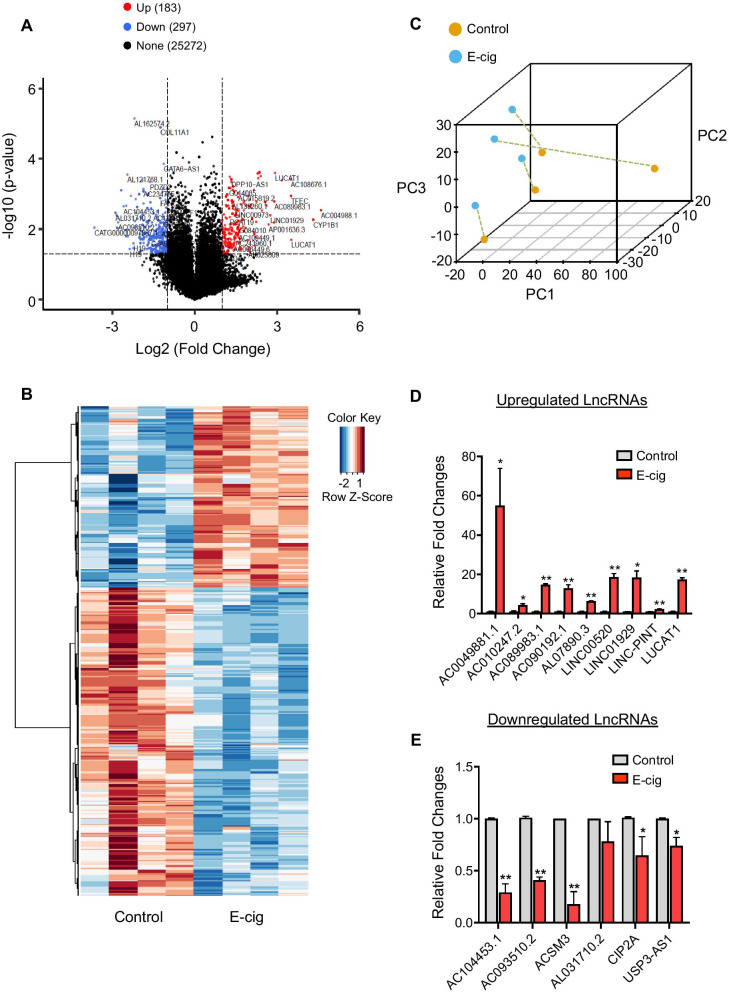
Differentially expressed lncRNAs in iPSC-ECs treated with e-cig. **A** Volcano plot of differentially expressed lncRNAs is shown, where red and blue dots represent lncRNAs that are up- and downregulated, respectively, with at least a fold change of 2 and *p* value < 0.05. **B** Hierarchical clustering analysis showing differentially expressed lncRNAs between non-treated (control) and treated groups with e-cig (6.5 TPE EAE). **C** PCA of lncRNA expression profiles is shown, with treated and untreated samples connected for each subject. A qPCR validation of **D** up- and **E** downregulated lncRNAs following EAE treatment (6.5 TPE). Data were obtained using iPSC-ECs from four healthy donors, and the assays were repeated three times. Data are represented as mean ± SD. * and ** indicate *p* < 0.05 and *p* < 0.001, respectively

To understand the potential roles of lncRNAs in regulating e-cig-induced endothelial dysfunction, we next investigated the genome-wide differential expression profiling of lncRNAs and mRNAs following e-cig treatment. An optimal concentration of EAE at 6.5 TPE was chosen which reduced cell viability by around 30% after 24 h exposure in vitro. To identify the transcripts that were dysregulated in ECs upon exposure to e-cig, we compared the expression profiles of lncRNAs and mRNAs in iPSC-ECs with or without e-cig exposure by performing microarray analysis. After checking the distribution of maximum, minimum, and percentile values of normalized signals for each sample (Additional file 1: Fig. S2), we found a total of 480 differentially expressed lncRNAs (Fig. 2A; 183 upregulated and 297 downregulated) with fold change ≥ 2.0 (Gene Expression Omnibus [GEO] ID code GSE186227). Hierarchical clustering was then performed to distinguish lncRNAs expression pattern between the control and e-cig-treated cells (Fig. 2B). The top 50 differentially expressed lncRNAs, up- and downregulated, are listed in Additional File 1: Table S2. Unsupervised principal component analysis (PCA) revealed that the lncRNA expression profiles of non-treated controls from four donors were closely clustered and separated from e-cig-treated samples (Fig. 2C). A total of 15 lncRNAs differentially expressed from Fig. 2B were selected for qPCR validation. Consistent with the data from the microarray, AC0049881.1, AC010247.2, AC089983.1, AC090192.1, AL07890.3, LINC00520, LINC01929, LINC-PINT, and LUCAT1 were found to be significantly upregulated (Fig. 2D), whereas AC104453.1, AC093510.2, ACSM3, CIP2A, and USP3-AS1 were found to be significantly downregulated following e-cig exposure (Fig. 2E).

**Fig. 3:**
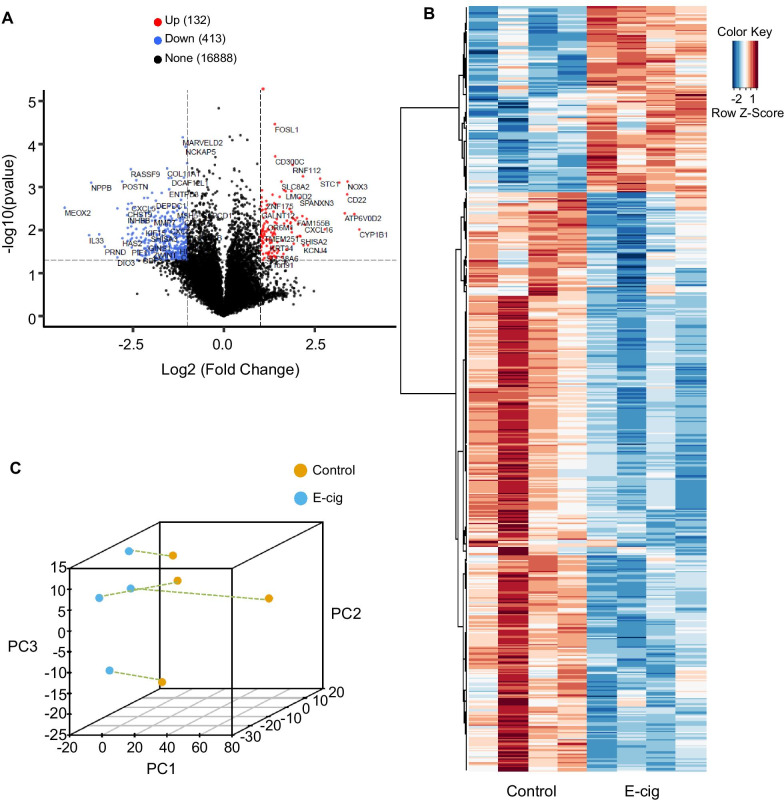
Differentially expressed mRNAs in iPSC-ECs treated with e-cig. **A** Volcano plot of differentially expressed mRNAs is shown, where red and blue dots represent genes that are up- and downregulated, respectively, with at least a fold change of 2 and *p* value < 0.05. **B** Hierarchical clustering analysis showing differentially expressed mRNAs between non-treated (control) and e-cig (6.5 TPE)-treated group. **C** PCA of mRNA expression profiles is shown, with treated and untreated samples connected for each subject

Microarray analysis also identified 545 differentially expressed mRNAs (Fig. 3A; 132 upregulated and 413 downregulated) with fold change ≥ 2.0. Hierarchical clustering was used to distinguish mRNAs expression pattern between the control and e-cig-treated groups (Fig. 3B) and the top 50 differentially expressed mRNAs, up- and downregulated, are listed in Additional file 1: Table S3. PCA was performed on the mRNA expression, and similar to the PCA done on lncRNA expression, there was clustering by samples with e-cig treatment (Fig. 3C).

### Gene Ontology (GO) and Kyoto Encyclopedia of Genes and Genome (KEGG) pathway analyses of differentially expressed mRNAs following e-cig exposure

**Fig. 4:**
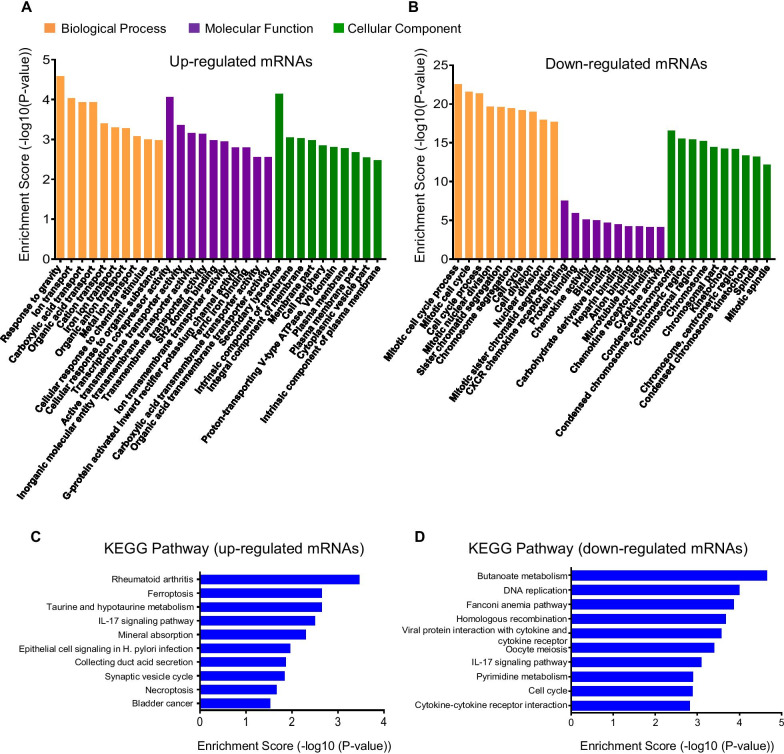
GO and KEGG pathway analyses of DEGs between e-cig treatment and controls. GO annotation of **A** up and **B** downregulated mRNAs with top 10 enrichment score covering domains of biological process (yellow), molecular function (purple), and cellular component (green). KEGG pathway enrichment analysis of **C** up and **D** downregulated mRNAs with the top 10 enrichment score

To further explore and categorize the biological functions of the 545 differentially expressed mRNAs, GO and KEGG enrichment analyses were conducted. The top 10 enriched GO terms in three domains including Biological Process (BP), Molecular Function (MF), and Cellular Component (CC) are shown in Fig. 4A, B (separate domains shown in Additional file 1: Fig. S3). For upregulated mRNAs, the top three GO processes included response to gravity (GO: 0009629), ion transport (GO: 0006811), and carboxylic acid transport (GO: 0015849) in BP; transcription corepressor activity (GO: 0003714), active transmembrane transporter activity (GO: 0022804), and inorganic molecular entity transmembrane transporter activity (GO: 0015318) in MF; secondary lysosome (GO: 0005767), intrinsic component of membrane (GO: 0031224), and integral component of membrane (GO: 0016021) in CC (Fig. 4A). In addition, top three GO processes for downregulated mRNAs included mitotic cell cycle process (GO: 1903047), mitotic cell cycle (GO:0000278), cell cycle process (GO: 0022402) in BP; CXCR chemokine receptor binding (GO: 0045236), protein binding (GO: 0005515), and chemokine activity (GO: 0008009) in MF; condensed chromosome (GO: 0000793), condensed chromosome, centromeric region (GO: 0000779), and chromosomal region (GO: 0098687) in CC (Fig. 4B).

The results in Fig. 4C, D represent the most vital KEGG pathways of the up- and downregulated differentially expressed genes (DEGs), respectively. When compared to controls, the upregulated mRNAs were primarily enriched in pathways associated with rheumatoid arthritis, ferroptosis, taurine and hypotaurine metabolism, IL-17 signaling pathway, mineral absorption, epithelial cell signaling in H. pylori infection, collecting duct acid secretion, synaptic vesicle cycle, necroptosis, and bladder cancer (Fig. 4C). By contrast, the downregulated DEGs were mainly responsible for butanoate metabolism, DNA replication, Fanconi anemia pathway, homologous recombination, viral protein interaction with cytokine and cytokine receptor, oocyte meiosis, IL-17 signaling pathway, pyrimidine metabolism, cell cycle, and cytokine-cytokine receptor interaction (Fig. 4D).

### Construction of lncRNA-mRNA co-expression network

**Fig. 5:**
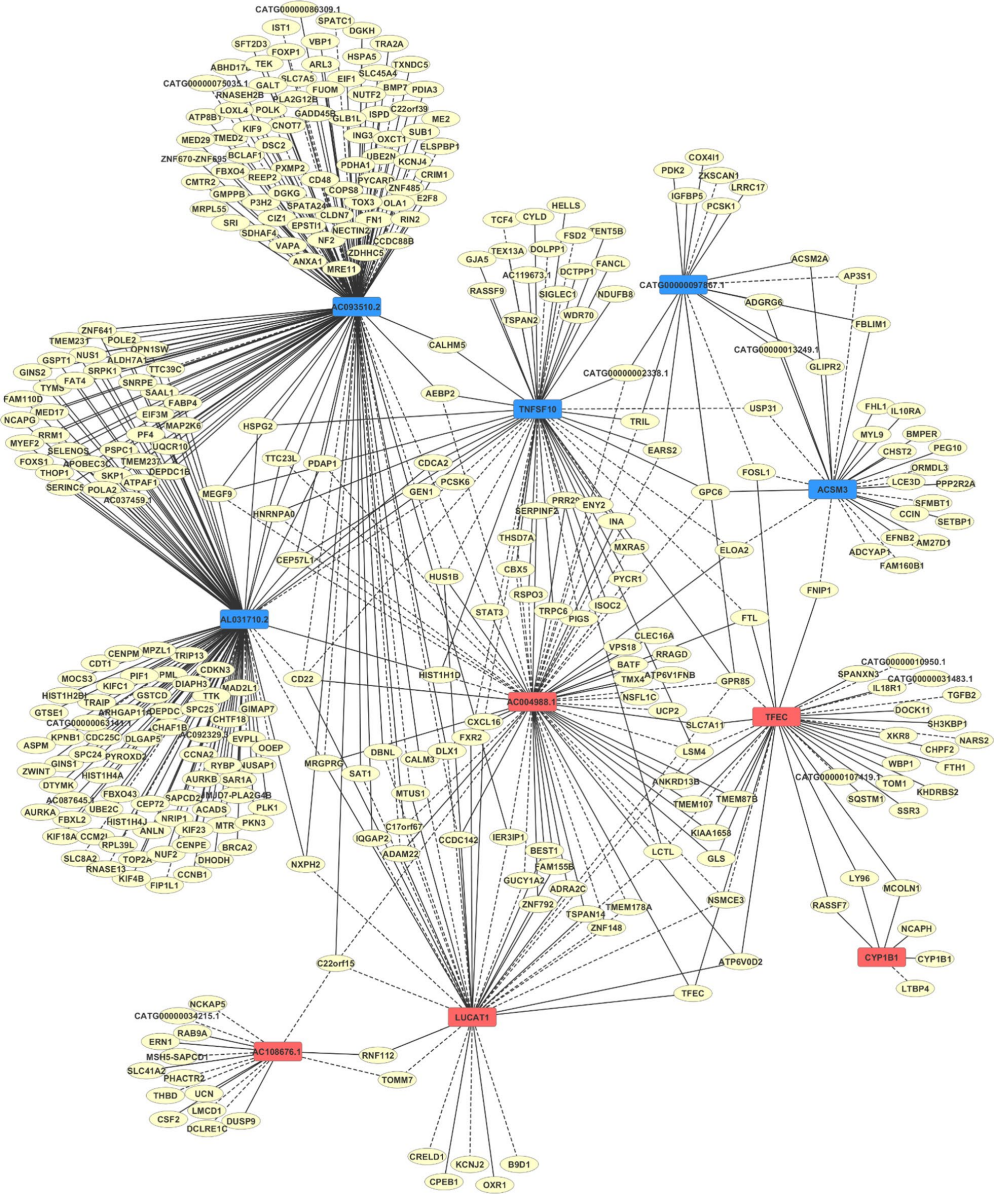
Correlation between top 5 significantly upregulated and downregulated lncRNAs and mRNA transcripts. LncRNA-mRNA co-expression network was constructed with top 5 up- and downregulated lncRNAs and 353 associated mRNAs. Red nodes, blue nodes, and yellow nodes correspond to upregulated lncRNAs, downregulated lncRNAs, and coding genes/mRNAs. Solid lines signify a positive Pearson’s correlation coefficient (PCC), and dotted lines signify negative PCC. Coding–noncoding pairs are defined as co-expressing pairs if ∣PCC∣  ≥ 0.9 and *p* ≤ 0.05

Additionally, significantly co-expressed lncRNA-mRNA pairs were identified via Pearson correlation coefficient (PCC) analysis (│PCC│ ≥ 0.9 and *p* ≤ 0.05) and assembled into a co-expression network to identify hub regulatory factors associated with e-cig exposure (Additional file 1: Table S4 and Additional file 1: Table S5). The top 5 up- and downregulated lncRNAs exhibiting the most significant differential expression (Table 1) were selected for constructing co-expression network to evaluate potential associations with mRNAs. Co-expression network analysis of these 5 upregulated lncRNAs (i.e., AC004988.1, CYP1B1, LUCAT1, TFEC, and AC108676.1) and 131 relevant mRNAs and downregulated 5 lncRNAs (i.e., CATG00000097867.1, AL031710.2, TNFSF10, ACSM3, and AC093510.2) and 281 associated mRNAs is shown in Fig. 5. The mRNAs associated with the 5 upregulated lncRNAs are correlated with binding, macromolecule modification, and cellular response to stimuli/signaling. All five lncRNAs were associated with genes related to regulation of apoptotic process (GO: 0042981). Additionally, in line with our data indicating e-cig results in an increase of ROS, 3 lncRNAs (AC004988.1, LUCAT1, and TFEC) had target genes related to cellular response to oxidative stress. Interestingly, the 5 lncRNAs which were downregulated after e-cig exposure are correlated with small molecule metabolic process, cell cycle process, and DNA repair. Specifically, target mRNAs for three lncRNAs (CATG00000097867.1, AL031710.2, and ACSM3) participate in the regulation of fatty acid biosynthetic process (GO0006633). For example, *Acsm2a* (acyl-CoA synthetase medium-chain family member 2A) and *Acsm3* are associated with fatty acid metabolic process by catalyzing fatty acid activation [32–34]. The regulation of fatty acid biosynthetic process role of these mRNAs suggests the potential importance of these lncRNAs in ECs exposed to e-cig.

**Table 1:** The top 5 upregulated and downregulated lncRNAs differentially expressed between iPSC-ECs treated with e-cig and non-treated cells

lncRNA	Regulation	Normalized intensity		Fold change	*p* value
		Control group	E-cig group		
AC004988.1	Up	2.55 ± 0.33	7.15 ± 0.76	24.37	0.003
CYP1B1	Up	4.01 ± 1.29	8.34 ± 1.29	20.07	0.005
LUCAT1	Up	3.23 ± 1.41	6.75 ± 0.58	11.48	0.020
TFEC	Up	3.60 ± 0.70	7.12 ± 0.19	11.41	0.001
AC108676.1	Up	3.18 ± 1.05	6.69 ± 0.78	11.35	0.0004
CATG00000097867.1	Down	7.80 ± 0.52	4.15 ± 1.48	12.53	0.009
AL031710.2	Down	7.70 ± 0.75	4.82 ± 0.44	7.39	0.003
TNFSF10	Down	6.95 ± 0.20	4.11 ± 0.75	7.15	0.004
ACSM3	Down	10.36 ± 0.59	7.53 ± 1.42	7.10	0.009
AC093510.2	Down	5.84 ± 0.78	3.04 ± 0.39	6.93	0.006

### Genes related to FAO and metabolism are dysregulated in ECs following exposure to e-cig

**Fig. 6:**
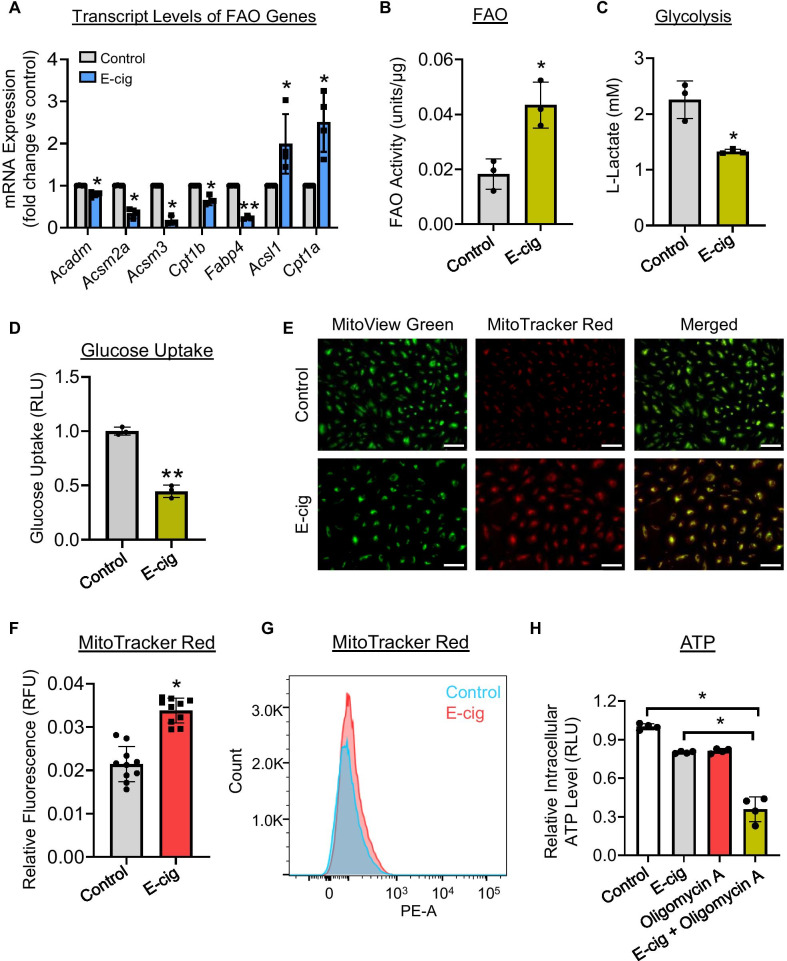
Assessment of metabolic changes in iPSC-ECs following e-cig treatment. **A** qPCR validation of FAO genes following e-cig treatment (6.5 TPE). **B** FAO, **C** glycolysis, and **D** glucose uptakes of iPSC-ECs following e-cig exposure (6.5 TPE) for 24 h (*n* = 3). Representative images and corresponding quantitative data of control and e-cig-treated cells taken with a fluorescence microscope (**E, F**) or flow cytometry (**G**). Live cells were stained with MitoTracker Red and MitoView Green. **H** ATP levels in cells treated with vehicle or e-cig (6.5 TPE) and/or 1.5 µM of oligomycin A for 48 h. Data are represented as mean ± SD, and * and ** indicate *p* < 0.05 and *p* < 0.001, respectively. Scale bars = 100 μm

As our co-expression network reveals that the target mRNAs for three of the top five lncRNAs downregulated following exposure to e-cig are responsible for fatty acid metabolism, transcript levels of FAO-related genes were first assessed (Fig. 6A and Additional file 1: Fig. S4). As shown in Fig. 6A, e-cig downregulated transcripts of various FAO genes including acyl-CoA dehydrogenase medium chain (*Acadm*), *Acsm2a*, *Acsm3*, carnitine palmitoyltransferase 1B (*Cpt1b*), and fatty acid binding protein 4 (*Fabp4*) while elevating acyl-coenzyme A synthetase long-chain family member 1 (*Acsl1*) and *Cpt1a*, which are essential enzymes responsible for carnitine shuttling.

As our data indicated e-cig impaired the genes responsible for fatty acid metabolism, we measured FAO activity, glycolysis, and glucose uptake to assess whether metabolic balance was altered in ECs following e-cig exposure. We found that FAO activity was significantly increased following 24 h of treatment with e-cig (Fig. 6B). Since ECs rely mostly on glycolysis and not fatty acid metabolism for energy, we then assessed whether glycolysis was perturbed and found that L-lactate concentration (Fig. 6C) and glucose uptake (Fig. 6D) were significantly decreased following 24-h e-cig exposure. As these data suggested that e-cig reduced the reliance on glycolysis for energy and activated aerobic metabolism, we then assessed mitochondrial activity in iPSC-ECs treated with e-cig. We stained iPSC-ECs with MitoTracker Red, which accumulation is dependent on the mitochondrial membrane potential. Flow cytometry and live cell imaging confirmed an increase in mitochondrial membrane potential in cells treated with e-cig for 48 h, compared to untreated cells (Fig. 6E–G); however, the change in membrane potential was not accompanied by changes in mitochondrial content as shown by MitoView green staining (Fig. 6E). Furthermore, 48-h treatment with e-cig caused a significant reduction in overall ATP levels of ECs and the reduced levels were further decreased when cells were co-treated with oligomycin A, an inhibitor of ATP synthase (Fig. 6H). Taken together, these results further suggested that EC metabolism is altered following e-cig treatment and the upregulation in FAO, which contributes minimally to ATP generation in ECs under basal conditions, compensates for e-cig-induced energy deficiency in ECs.

### LncRNA LUCAT1 regulates endothelial function

**Fig. 7:**
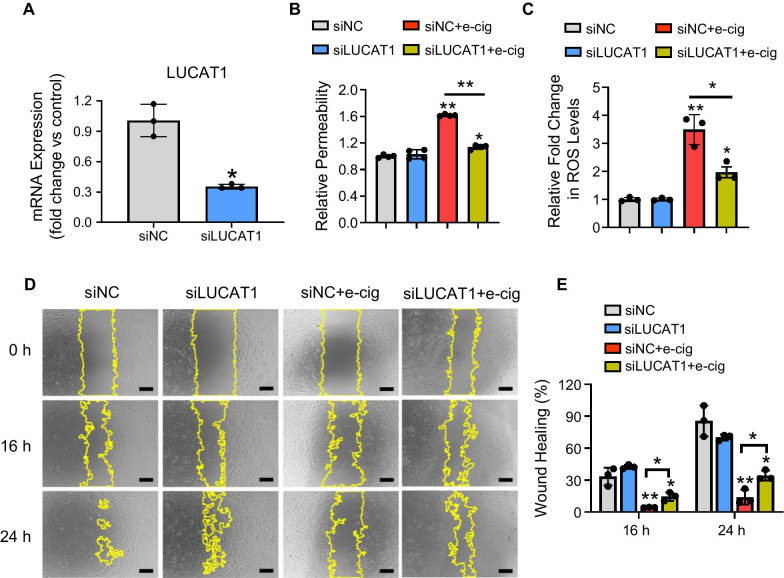
Assessment of the role of LUCAT1 in iPSC-ECs following e-cig treatment. **A** qPCR validation of LUCAT1 expression following knockdown with 25 µM siRNA for negative control (siNC) and for LUCAT1 (siLUCAT1). **B** Permeability, **C** ROS levels, and **D**, **E** representative images and corresponding quantitative data of migration assays were assessed in iPSC-ECs treated with either siNC, siLUCAT1, siNC + e-cig (6.5 TPE), or siLUCAT1 + e-cig (*n* = 3–4). Data are represented as mean ± SD, and * and ** indicate *p* < 0.05 and *p* < 0.001, respectively. Scale bars = 500 μm

As our data indicate that lncRNA LUCAT1 is one of the significantly upregulated lncRNAs after e-cig exposure (Fig. 2), we further assessed the role of LUCAT1 in EC phenotypes following e-cig treatment. We found that LUCAT1 knockdown using siRNA (Fig. 7A) did not affect cell viability after e-cig treatment (data not shown) but led to an attenuation of increased permeability (Fig. 7B) and ROS production (Fig. 7C) in iPSC-ECs caused by e-cig. Similarly, the e-cig-mediated reduction in migration ability of the iPSC-ECs was partially restored with LUCAT1 knockdown (Fig. 7D, E). Altogether, the results suggest that LUCAT1 plays a critical role in e-cig-induced endothelial dysfunction.

## Discussion

Recent studies have highlighted an emerging role of lncRNAs in regulating vascular function and remodeling [35, 36], and exosomal lncRNAs have been shown to be altered in e-cig user [37]; however, there is limited knowledge of the regulatory lncRNAs and their roles in ECs exposed to e-cig. In the current study, we performed a comprehensive analysis of differentially regulated lncRNAs and mRNAs by assessing the transcriptome profiles of human iPSC-ECs following e-cig exposure. Using hiPSC-ECs, we first exposed them to varying ranges of EAE and observed e-cig led to reduced cell viability, increased ROS generation and caspase 3/7 activity, and a reduction in a tube formation, migration ability, and barrier function, which are consistent with our previous study [11] demonstrating that e-cig exposure results in endothelial dysfunction. In addition, we successfully identified a total of 480 lncRNAs and 545 mRNAs which were differentially expressed between the two groups, controls and e-cig-treated samples (fold change ≥ 2, *p* < 0.05) and summarized their general characteristics and functional annotations revealing some of the potential functions and pathways related to the pathogenesis of e-cig-associated vascular diseases. We also showed that e-cig treatment led to metabolic alterations in ECs supported by an increase in FAO and mitochondrial membrane potential as well as a decrease in glycolysis and glucose uptake.

By utilizing GO and KEGG pathway analyses, we identified the biological functions of differentially expressed mRNAs and improved our understanding of the mechanisms of how e-cig affects ECs. We found that the commonly upregulated DEGs were highly enriched in cellular response to chemical stimulus [8] and ion transport [38] and these genes were further identified to be primarily related to pathways related to rheumatoid arthritis, inflammatory disorder leading to the activation of ECs, and ferroptosis, a regulated necrosis with dependency of iron and lipotoxicity [39]. Consistent with our finding, recent studies showed that ferroptosis plays a critical role in cigarette smoking-associated cytotoxicity in vascular smooth muscle cells [40] and lung epithelial cells [41]. In contrast, the genes involved in cell cycle progression and cell division [19, 20, 42] were persistently downregulated in ECs treated with e-cig, suggesting some inhibitory effects of the toxic compounds of e-cig in endothelial cell cycle progression and cell proliferation. In addition, analysis of the nature of these downregulated genes indicates that butanoate metabolism and DNA replication pathway were among the pathways with the most significantly enriched, which may reflect biological responses in ECs to e-cig components such as nicotine, ethyl butyrate, and ethyl alcohol and suggest many pathways related with cell cycle and DNA repair/replication were responsive to the e-cig exposure.

Although lncRNAs play critical regulatory roles via a variety of mechanisms, such as chromatin modification, transcriptional activation, transcriptional interference, RNA processing, and mRNA translation [43], the majority of them have unknown function. The dysregulated lncRNAs identified in this study will help to better understand the biological responses to e-cigs which, to date, have been left largely unexplored. Furthermore, lncRNA–mRNA co-expression network was explored to identify hub lncRNAs associated with e-cig exposure and the results revealed certain key downregulated lncRNAs, such as AC093510.2 and AL031710.2, and upregulated lncRNAs, such as AC004988.1 and LUCAT1. Interestingly, these downregulated lncRNAs were associated with genes participating in fatty acid metabolism, cell cycle, cell division, and cell adhesion, and upregulated lncRNAs were associated with genes involved in iron-ion binding, protein binding, and proton-transporting ATPase activity.

Despite their proximity to oxygenated blood, ECs predominantly use glucose as an energy source and utilize glycolysis instead of oxidative metabolism to generate 80% of their ATP [44]. In fact, less than 1% of glucose-derived pyruvates are oxidized through the tricarboxylic acid (TCA) cycle in the mitochondria to produce ATP [44]. However, when glycolysis is compromised or under stress conditions, ECs retain their capacity for oxidative metabolism via catabolism of glucose, fatty acids, and amino acids [45]. Cigarette smoke exposure resulted in inhibition of glycolysis in alveolar type II cells [22]. Acute cigarette smoke exposure also induced a switch of the main energy source from glucose to lipid and an increase of FAO in human bronchial epithelial cells [21]. In contrast, FAO was downregulated in CSE-treated mouse pulmonary microvascular ECs, which was linked to reduced *Cpt1a* [20]. This discrepancy may be attributable to differences in cell types as well as experimental conditions such as CS exposure duration. Importantly, whether e-cig causes metabolic dysfunction in ECs remain elusive. We found that e-cig exposure significantly reduced levels of most genes related to FAO. Interestingly, *Cpt1a*, the enzyme responsible for the rate-determining step of FAO, was found to be significantly upregulated. Consistent with *Cpt1a* expression, FAO was found to be significantly increased in iPSC-ECs treated with e-cig. This metabolic change was coupled with a downregulation of glycolysis and glucose uptake and activation of the mitochondria in ECs, suggesting that the FAO might be activated to compensate for the loss of the major energy source for ECs.

LUCAT1 was firstly reported in the airway epithelium of cigarette smokers and various lung cancer cell lines with conflicting roles. Its transcription is regulated by nuclear factor erythroid 2-related factor 2 (NRF2), which is implicated in cell survival through the regulation of ROS level, mediating a protective state in airway epithelial cells treated with CSE [17]. It has also been shown that the overexpression of LUCAT1 inhibits inducible nitric oxide synthase in lung cells under hyperglycemic conditions [46]. In line with our study, LUCAT1 was significantly increased in human bronchial epithelial cells following CSE treatment and the knockdown of LUCAT1 in conjunction with CSE treatment led to the alleviation of increased apoptosis and decreased cell proliferation caused by CSE [47]. Further, Zhao et al. demonstrated that these effects are due in part to LUCAT1’s ability to sponge miR-181a, inhibiting miR-181a’s silencing effects on the Wnt/β-catenin pathway [47]. Altogether, these previous studies suggest an essential role of LUCAT1 in regulating endothelial function.

This study has a few limitations that should be addressed in future studies. Our study relies on microarrays and thus is subjected to the limits of this assay covering only known lncRNAs. Therefore, novel lncRNAs may be missed in this study. Further, it should be noted that we did not sort live cells for the transcriptomic analysis as we sought to provide a transcriptome snapshot of the entire population of cells exposed to e-cig. Hence, there is a possibility that the gene expression changes seen partially reflect the survival effects of remaining cells. Future transcriptomic analysis either at lower TPE or earlier time points will help to better distinguish gene expression changes resulting directly because of e-cig toxicity as opposed to those occurring due to changes in survival rates. Although we identified several lncRNAs and pathways associated with endothelial dysfunction following e-cig exposure, it is imperative to further characterize the molecular mechanisms of these potential regulators in ECs to develop their therapeutic potentials. Furthermore, our study utilized one e-cig device and e-liquid with analysis performed after an acute exposure. As e-cigs are diverse in components (e.g., PG/VG and nicotine) and flavorings including tobacco and mint/menthol or cooling agents, and usage can vary among users, our study may not recapitulate the experience of the typical e-cig user. Further studies are required to determine the effects of various e-cig components including unregulated tobacco and/or mint/menthol flavors and flavoring chemicals on regulation of lncRNA in iPSC-ECs.

## Conclusions

The present study provides an expression profile of differentially expressed lncRNAs and mRNAs in iPSC-ECs after e-cig treatment. A comprehensive bioinformatic analysis of DEGs revealed several potential regulators and pathways that will provide a new perspective on the mechanisms involved in e-cig-induced EC dysfunction, which is conducive for future investigation into novel diagnostic and therapeutic strategies.

## Supplementary Information

**Additional file 1.** Fig. S1. In vitro characterization of hiPSC-ECs. A Schematic illustration of endothelial differentiation protocol of iPSCs. B Flow cytometry analysis of iPSC-ECs was utilized to assess EC differentiation efficiency. iPSC-ECs were stained with endothelial markers, such as VE-cadherin (CD144) and PECAM1 (CD31), a hematopoietic marker (CD45), and a progenitor marker (CD34). C Immunofluorescence staining of endothelial markers, such as acetylated low-density lipoprotein (Ac-LDL) and VE-cadherin (VE-CAD), was performed on iPSC-ECs. Scale bars = 100 μm. Fig. S2. Box plots showing normalized intensity of each sample for A lncRNAs and B mRNAs probed by microarray. Fig. S3. The Go terms were divided into three categories, including biological process (BP, yellow), molecular function (MF, purple), and cellular component (CC, green). Top 10 significantly up- and downregulated GO terms for differentially expressed mRNAs. Fig. S4. Expression of fatty acid oxidation-related genes. Expression of acetyl-CoA acyltransferase 1 (*Acaa1*), acyl-CoA synthetase long-chain family member 3 (*Acsl3*), and hydroxyacyl-CoA dehydrogenase trifunctional multienzyme complex subunit alpha (*Hadha*) following EAE exposure (6.5 TPE) was quantified using qPCR. Data are represented as mean ± SD. Table S1. PCR primers used for validation studies. Primers are indicated as forward (F) or reverse (R). Table S2. Top 50 differentially expressed lncRNAs regulated by e-cig. Table S3. Top 50 differentially expressed mRNAs regulated by e-cig. Table S4. mRNA–lncRNA pairs identified in the expression network for upregulated lncRNAs. Table S5. mRNA–lncRNA pairs identified in the expression network for downregulated lncRNAs.

## Data Availability

Data generated or analyzed during this study are available from the corresponding author on request.

## Abbreviations

CSE: Cigarette smoke extract
E-cig: Electronic cigarette
EAE: E-cig aerosol extract
ECs: Endothelial cells
ENDS: Electronic nicotine delivering system
FAO: Fatty acid oxidation
iPSC-ECs: Induced pluripotent stem cell-derived endothelial cells
iPSC: Induced pluripotent stem cells
LDL: Low-density lipoprotein
LncRNAs: Long noncoding RNAs
PG: Propylene glycol
TPE: Typical puff equivalent
VG: Vegetable glycerin
