# Supplementary material for: Genome-wide differential expression profiling of lncRNAs and mRNAs in human induced pluripotent stem cell-derived endothelial cells exposed to e-cigarette extract
Source: Stem Cell Res Ther. 2021 Dec 4;12:593. doi: 10.1186/s13287-021-02654-6 (PMC8643021; doi:10.1186/s13287-021-02654-6)
Supplement: Supplementary file 1 — Additional file 1. Fig. S1. In vitro characterization of hiPSC-ECs. A Schematic illustration of endothelial differentiation protocol of iPSCs. B Flow cytometry analysis of iPSC-ECs was utilized to assess EC differentiation efficiency. iPSC-ECs were stained with endothelial markers, such as VE-cadherin (CD144) and PECAM1 (CD31), a hematopoietic marker (CD45), and a progenitor marker (CD34). C Immunofluorescence staining of endothelial markers, such as acetylated low-density lipoprotein (Ac-LDL) and VE-cadherin (VE-CAD), was performed on iPSC-ECs. Scale bars = 100 μm. Fig. S2. Box plots showing normalized intensity of each sample for A lncRNAs and B mRNAs probed by microarray. Fig. S3. The Go terms were divided into three categories, including biological process (BP, yellow), molecular function (MF, purple), and cellular component (CC, green). Top 10 significantly up- and downregulated GO terms for differentially expressed mRNAs. Fig. S4. Expression of fatty acid oxidation-related genes. Expression of acetyl-CoA acyltransferase 1 (Acaa1), acyl-CoA synthetase long-chain family member 3 (Acsl3), and hydroxyacyl-CoA dehydrogenase trifunctional multienzyme complex subunit alpha (Hadha) following EAE exposure (6.5 TPE) was quantified using qPCR. Data are represented as mean ± SD. Table S1. PCR primers used for validation studies. Primers are indicated as forward (F) or reverse (R). Table S2. Top 50 differentially expressed lncRNAs regulated by e-cig. Table S3. Top 50 differentially expressed mRNAs regulated by e-cig. Table S4. mRNA–lncRNA pairs identified in the expression network for upregulated lncRNAs. Table S5. mRNA–lncRNA pairs identified in the expression network for downregulated lncRNAs. [file 13287_2021_2654_MOESM1_ESM.docx]

**Supplemental File 1**

**Genome-wide Differential Expression Profiling of lncRNAs and mRNAs in Human Induced Pluripotent Stem Cell-Derived Endothelial Cells Exposed to E-cigarette Extract**

Hoai Huong Thi Le^1^, Chen-wei Liu^1^, Philip Denaro III^1^, Jordan Jousma^2^, Ning-Yi Shao^3^,

Irfan Rahman^4^, and Won Hee Lee^1,*^

^1^Department of Basic Medical Sciences, University of Arizona College of Medicine, Phoenix, Arizona, 85004, USA; ^2^Department of Pharmacology & Regenerative Medicine, University of Illinois College of Medicine, Chicago, Illinois, 60612, USA; ^3^Health Sciences, University of Macau, Macau, China; ^4^Department of Environmental Medicine, University of Rochester Medical Center, Rochester, New York, 14642, USA

**
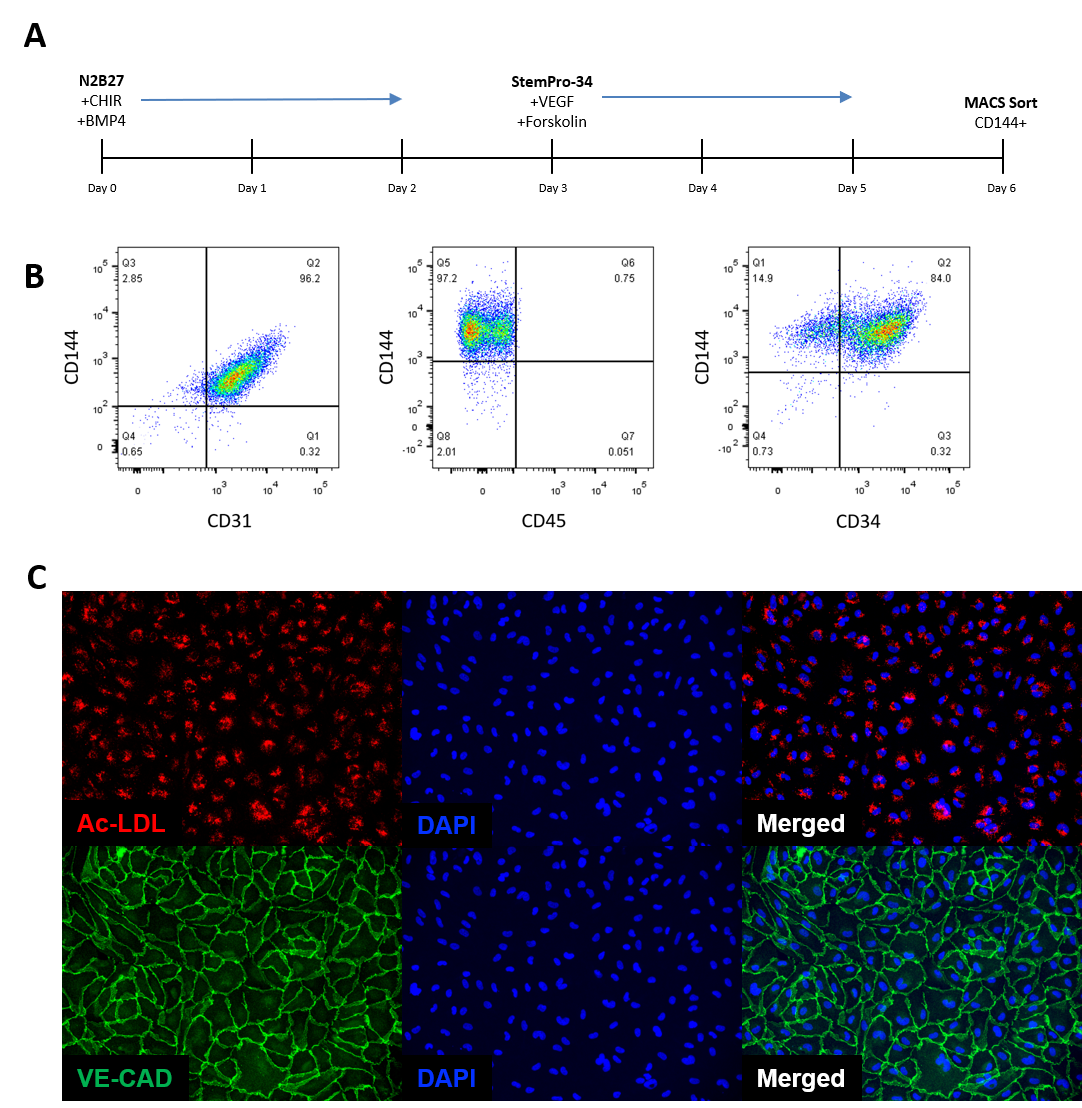
**

**
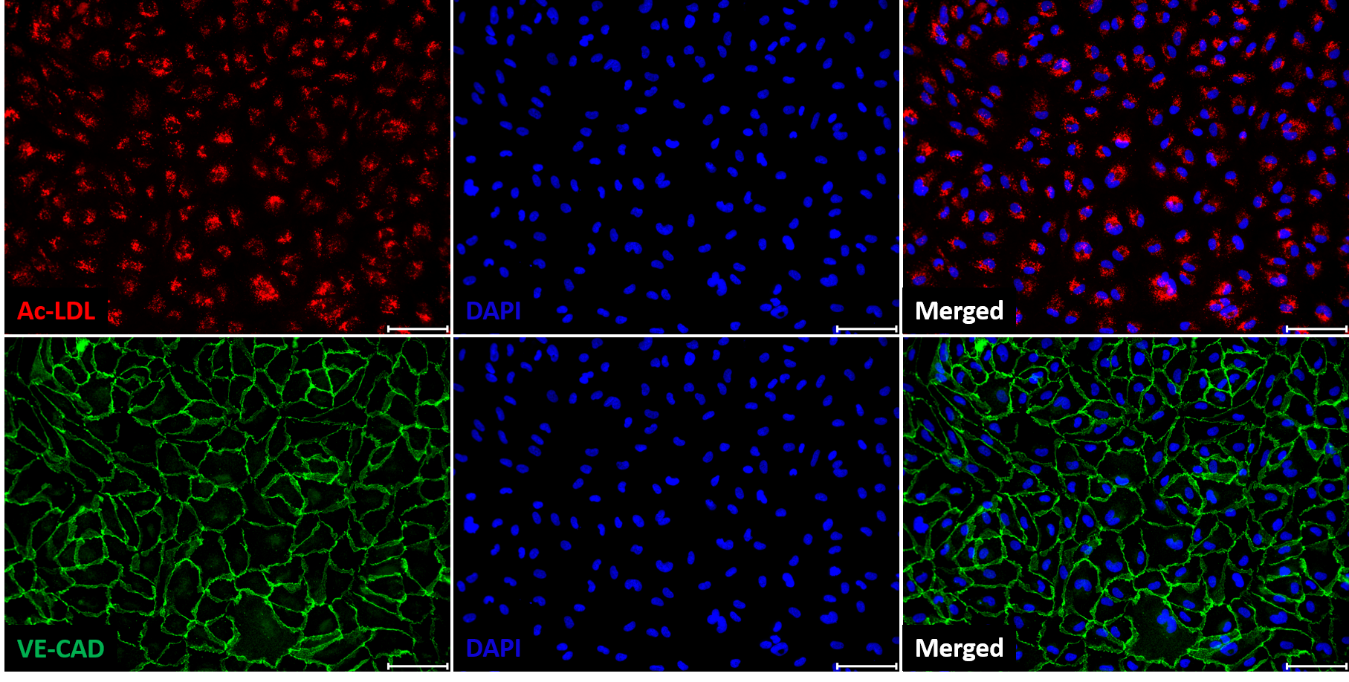
**

**Fig. S1**. *In vitro* characterization of hiPSC-ECs. **(A)** Schematic illustration of endothelial differentiation protocol of iPSCs. **(B)** Flow cytometry analysis of iPSC-ECs was utilized to assess EC differentiation efficiency. iPSC-ECs were stained with endothelial markers, such as VE-cadherin (CD144) and PECAM1 (CD31), a hemopoietic marker (CD45), and a progenitor marker (CD34). **(C)** Immunofluorescence staining of endothelial markers, such as acetylated-low density lipoprotein (Ac-LDL) and VE-cadherin (VE-CAD) was performed on iPSC-ECs. Scale bars = 100 μm.


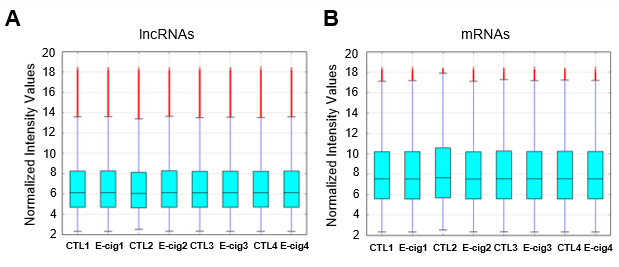


**Fig. S2**. Box plots showing normalized intensity of each sample for **(A)** lncRNAs and **(B)** mRNAs probed by microarray.

**
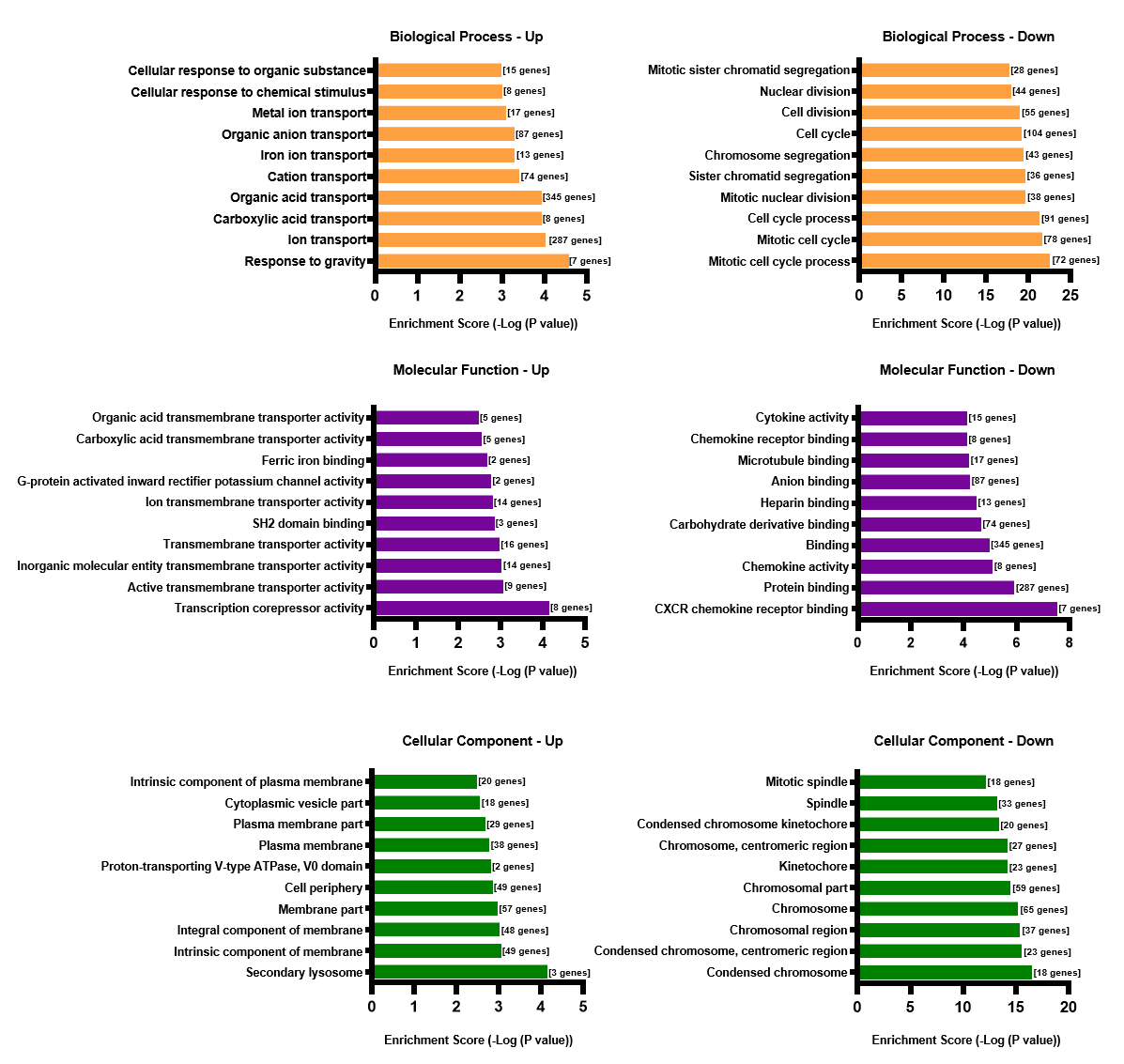
**

**Fig. S3.** The Go terms were divided into three categories, including biological process (BP, yellow), molecular function (MF, purple), and cellular component (CC, green). Top 10 significantly up- and down-regulated GO terms for differentially expressed mRNAs.

**mRNA expression**

**(fold change vs control)**

**Fig. S4. Expression of fatty acid oxidation-related genes.**  Expression of acetyl-CoA acyltransferase 1(*Acaa1*), acyl-CoA synthetase long chain family member 3 (*Acsl3*), and Hydroxyacyl-CoA Dehydrogenase Trifunctional Multienzyme Complex Subunit Alpha (*Hadha*) following EAE exposure (6.5 TPE) was quantified using qPCR. Data are represented as mean ± SD.

**Table S1**. PCR primers used for validation studies. Primers are indicated as forward (F) or reverse (R).

| **Gene Symbol** | **Sequence Name** | **Primer Sequence/ ID No.** |
| --- | --- | --- |
| AC093510.2 | ENST00000562820 | F: CCCGGAGTCCAACATTTCT  R: GGCAGTCTAGGTAACACCATAC |
| AC104453.1 | ENST00000648602 | F: CTGGAAACATAAGCGCTTTCAG  R: GTGTGTGAGAGAGAGAGAGAGA |
| ACSM3 | ENST00000614721 | F: CAGCCAGTGTTTGGTTGTTAAA  R: GTTGCCCTAGGCAGGTTATT |
| AL031710.2 | ENST00000621884 | F: GAGTTTCTGGAGGTGATGTCTT  R: CAGAAGCCAGAGTGGAGTTT |
| CIPA2 | ENST00000487834 | F: TCAGACGAGGGTGGGTTAG  R: GGGACCGTTCTCGGTGA |
| USP3-AS1 | ENST00000561256 | F: AGGTGATTTCGGTCCAACG  R: TCAACACCACCGCCTTTC |
| AC0049881.1 |  | BioRad UniqueID: qhsaLID0071158 |
| AC010247.2 |  | BioRad UniqueID: qhsaLED0106278 |
| AC089983.1 |  | BioRad UniqueID: qhsaLID0071053 |
| AC090192.2 |  | BioRad UniqueID: qhsaLED0062724 |
| AL078590.3 |  | BioRad UniqueID: qhsaLED0216177 |
| LINC00520 |  | BioRad UniqueID: qhsaLED0214735 |
| LINC01929 |  | BioRad UniqueID: qhsaLID0060458 |
| LINC-PINT |  | F: ACAAATCTACGTGCGCATCA  R: AGCAAGGCAGAGAAACTCCA |
| LUCAT1 |  | F: GCTCGGATTGCCTTAGACAG  R: GGGTGAGCTTCTTGTGAGGA |
| GAPDH |  | F:TCAAGGCTGAGAACGGGAAG  R:GGACTCCACGACGTACTCAG |
| ACAA1 |  | F: GACAGGTCATCACGCTGCTCAA  R: CCAGGGTATTCAAAGACGGCAG |
| ACADM |  | F: AGAACCTGGAGCAGGCTCTGAT  R: GGATCTGGATCAGAACGTGCCA |
| ACSL1 |  | F: ATCAGGCTGCTCATGGATGACC  R: AGTCCAAGAGCCATCGCTTCAG |
| ACSL3 |  | F: CTTTCTCACGGATGCCGCATTG  R: CTGCTGCCATCAGTGTTGGTTTC |
| ACSM2A |  | F: CAGAAGGAGACATTGGCATCAGG  R: AGTCTCCTCGAATGTTGGCTGC |
| ACSM3 |  | F: CTGGGCAAAGTCTGCATGGAGT  R: AGTTGGTGCTGAACAGAAGACTG |
| CPT1A |  | F: GATCCTGGACAATACCTCGGAG  R: CTCCACAGCATCAAGAGACTGC |
| CPT1B |  | F: TGTATCGCCGTAAACTGGACCG  R: TGTCTGAGAGGTGCTGTAGCAC |
| FABP4 |  | F: ACGAGAGGATGATAAACTGGTGG  R: GCGAACTTCAGTCCAGGTCAAC |
| HADHA |  | F: GCCGACATGGTGATTGAAGCTG  R: GGAGAGCAGATGTGTTACTGGC |

**Table S2.** Top 50 differentially expressed lncRNAs regulated by e-cig.

| **Transcript ID** | **Gene Symbol** | **Regulation** | **Fold Change** | **P-value** |
| --- | --- | --- | --- | --- |
| ENST00000415237 | AC004988.1 | up | 24.37 | 0.003 |
| ENST00000491456 | CYP1B1 | up | 20.07 | 0.005 |
| ENST00000649702 | LUCAT1 | up | 11.48 | 0.020 |
| ENST00000497829 | TFEC | up | 11.41 | 0.001 |
| ENST00000455557 | AC108676.1 | up | 11.35 | 0.000 |
| ENST00000517482 | AC090192.2 | up | 9.09 | 0.000 |
| ENST00000549807 | AC089983.1 | up | 7.48 | 0.002 |
| ENST00000560267 | LINC00520 | up | 7.40 | 0.007 |
| ENST00000589125 | LINC01929 | up | 6.76 | 0.004 |
| ENST00000525714 | AP001636.3 | up | 6.50 | 0.007 |
| ENST00000552324 | AC084032.1 | up | 6.14 | 0.002 |
| T183149 | G042041 | up | 5.94 | 0.002 |
| ENST00000620908 | AL121829.2 | up | 5.45 | 0.003 |
| ENST00000423028 | AL078590.3 | up | 5.30 | 0.000 |
| ENST00000609238 | AP005137.2 | up | 5.13 | 0.000 |
| ENST00000647102 | AC016831.6 | up | 4.91 | 0.000 |
| T363698 | G085916 | up | 4.77 | 0.002 |
| ENST00000647202 | AC005280.2 | up | 4.74 | 0.006 |
| ENST00000416908 | AL391811.1 | up | 4.72 | 0.017 |
| ENST00000510996 | AC097372.1 | up | 4.58 | 0.029 |
| ENST00000559786 | AC010247.2 | up | 4.53 | 0.014 |
| ENST00000443623 | LINC-PINT | up | 4.45 | 0.012 |
| ENST00000607528 | AL357078.3 | up | 4.28 | 0.005 |
| T175957 | G040741 | up | 4.18 | 0.002 |
| ENST00000553682 | LINC01269 | up | 4.10 | 0.002 |
| ENST00000508827 | LINC01303 | up | 4.05 | 0.001 |
|  |  |  |  |  |

**Table S2.** (Continued).

| **Transcript ID** | **Gene Symbol** | **Regulation** | **Fold Change** | **P-value** |
| --- | --- | --- | --- | --- |
| T185828 | G042655 | up | 4.04 | 0.044 |
| ENST00000624941 | AC079298.3 | up | 3.97 | 0.032 |
| ENST00000562696 | AC092115.2 | up | 3.92 | 0.002 |
| uc001eqi.1 | AK023809 | up | 3.88 | 0.038 |
| ENST00000430078 | AL078604.2 | up | 3.86 | 0.001 |
| ENST00000424493 | AC024560.1 | up | 3.62 | 0.002 |
| ENST00000606343 | AL365181.2 | up | 3.61 | 0.009 |
| BIG-lncRNA-572.1_CPS.44855779 | BIG-lncRNA-572 | up | 3.51 | 0.002 |
| ENST00000453639 | AL355314.1 | up | 3.47 | 0.011 |
| ENST00000454600 | LINC01524 | up | 3.46 | 0.003 |
| TCONS_00003694 | XLOC_001484 | up | 3.44 | 0.002 |
| ENST00000650414 | AC244021.1 | up | 3.40 | 0.008 |
| ENST00000429998 | RP11-513I15.6 | up | 3.36 | 0.005 |
| ENST00000538682 | SLC3A2 | up | 3.35 | 0.005 |
| TCONS_00024443 | XLOC_011766 | up | 3.23 | 0.010 |
| ENST00000589379 | AC011511.5 | up | 3.23 | 0.037 |
| ENST00000438824 | LINC01802 | up | 3.19 | 0.002 |
| ENST00000511928 | LINC02600 | up | 3.18 | 0.019 |
| ENST00000412162 | MIR181A1HG | up | 3.15 | 0.033 |
| ENST00000602554 | AC011450.1 | up | 3.15 | 0.038 |
| ENST00000472890 | LINC01213 | up | 3.12 | 0.024 |
| NR_119377 | DLGAP1-AS2 | up | 3.10 | 0.011 |
| compmerge.2661.pooled.chr11 | RAB30-AS1 | up | 3.09 | 0.018 |
| T356261 | G084010 | up | 3.07 | 0.008 |
| ENCT00000421817 | CATG00000097867.1 | down | 12.53 | 0.009 |
| ENST00000621884 | AL031710.2 | down | 7.39 | 0.003 |

**Table S2.** (Continued).

| **Transcript ID** | **Gene Symbol** | **Regulation** | **Fold Change** | **P-value** |
| --- | --- | --- | --- | --- |
| NR_033994 | TNFSF10 | down | 7.15 | 0.004 |
| ENST00000614721 | ACSM3 | down | 7.10 | 0.009 |
| ENST00000562820 | AC093510.2 | down | 6.93 | 0.006 |
| ENST00000625474 | TGFB2-OT1 | down | 6.42 | 0.001 |
| ENST00000648602 | AC104453.1 | down | 6.05 | 0.002 |
| ENST00000610230 | AL360219.1 | down | 5.66 | 0.016 |
| ENST00000413991 | AC073257.2 | down | 5.64 | 0.002 |
| ENST00000604271 | AC007681.1 | down | 5.64 | 0.011 |
| ENST00000613917 | AL121768.1 | down | 5.47 | 0.000 |
| uc003nhj.3 | BC079832 | down | 5.42 | 0.016 |
| ENST00000412788 | H19 | down | 5.16 | 0.037 |
| ENST00000522354 | AC083967.1 | down | 5.02 | 0.001 |
| ENST00000415479 | LINC01614 | down | 4.73 | 0.035 |
| ENST00000614618 | AL162574.2 | down | 4.61 | 0.000 |
| ENST00000613987 | AC020658.4 | down | 4.55 | 0.006 |
| ENST00000445932 | AL451164.2 | down | 4.51 | 0.009 |
| NR_034078 | LOC643733 | down | 4.48 | 0.008 |
| T297462 | G069497 | down | 4.45 | 0.001 |
| ENST00000547898 | AC079600.3 | down | 4.29 | 0.002 |
| T046285 | G010780 | down | 4.23 | 0.005 |
| ENST00000437601 | LINC01132 | down | 4.08 | 0.017 |
| ENST00000512512 | IGFBP7 | down | 4.07 | 0.001 |
| ENST00000619110 | AL158163.2 | down | 4.05 | 0.045 |
| T162082 | G037470 | down | 4.02 | 0.017 |
| ENST00000620080 | AL023803.2 | down | 3.99 | 0.009 |
| NR_026991 | H1FX-AS1 | down | 3.99 | 0.014 |
|  |  |  |  |  |

**Table S2.** (Continued).

| **Transcript ID** | **Gene Symbol** | **Regulation** | **Fold Change** | **P-value** |
| --- | --- | --- | --- | --- |
| HBMT00000723185 | CATG00000039975.1 | down | 3.95 | 0.018 |
| ENST00000614337 | KNL1 | down | 3.94 | 0.025 |
| T371403 | G087584 | down | 3.91 | 0.040 |
| ENST00000605067 | GFOD1 | down | 3.83 | 0.041 |
| ENST00000557813 | CGNL1 | down | 3.82 | 0.005 |
| ENST00000482019 | LINC02004 | down | 3.79 | 0.004 |
| NR_037599 | PDCD6IPP2 | down | 3.78 | 0.012 |
| ENST00000450746 | LINC01985 | down | 3.75 | 0.012 |
| ENST00000462959 | AC016747.4 | down | 3.74 | 0.008 |
| ENST00000608444 | AC016394.1 | down | 3.73 | 0.019 |
| ENST00000608152 | LAMTOR5-AS1 | down | 3.73 | 0.000 |
| ENST00000421318 | AL356277.2 | down | 3.72 | 0.015 |
| ENST00000555377 | AL359232.1 | down | 3.72 | 0.014 |
| ENST00000216414 | CDKN3 | down | 3.66 | 0.001 |
| ENST00000564612 | AC234775.3 | down | 3.65 | 0.001 |
| ENST00000597278 | AL009178.2 | down | 3.65 | 0.006 |
| HBMT00000760533 | CATG00000042175.1 | down | 3.64 | 0.002 |
| ENST00000444114 | LINC01638 | down | 3.63 | 0.025 |
| ENST00000585816 | AC011447.3 | down | 3.63 | 0.019 |
| ENST00000589892 | BIRC5 | down | 3.61 | 0.017 |
| ENST00000544931 | RAD51AP1 | down | 3.57 | 0.013 |
| ENST00000548946 | UBE2N | down | 3.50 | 0.009 |

**Table S3.** Top 50 differentially expressed mRNAs regulated by e-cig.

| **Sequence Name** | **Gene Symbol** | **Regulation** | **Fold Change** | **P-value** |
| --- | --- | --- | --- | --- |
| ENST00000610745 | CYP1B1 | up | 13.21 | 0.010 |
| ENST00000369415 | RRAGD | up | 12.00 | 0.004 |
| ENST00000159060 | NOX3 | up | 10.55 | 0.001 |
| ENST00000085219 | CD22 | up | 10.49 | 0.001 |
| ENST00000285393 | ATP6V0D2 | up | 10.01 | 0.004 |
| ENST00000265440 | TFEC | up | 8.99 | 0.001 |
| ENST00000262593 | DOK5 | up | 6.82 | 0.010 |
| ENST00000290271 | STC1 | up | 6.26 | 0.001 |
| ENST00000216492 | CHGA | up | 4.96 | 0.022 |
| ENST00000399298 | TMEM26 | up | 4.84 | 0.005 |
| ENST00000293778 | CXCL16 | up | 4.65 | 0.007 |
| ENST00000303592 | KCNJ4 | up | 4.55 | 0.023 |
| ENST00000280612 | SLC7A11 | up | 4.51 | 0.001 |
| ENST00000281156 | KHDRBS2 | up | 4.46 | 0.005 |
| ENST00000319420 | SHISA2 | up | 4.30 | 0.014 |
| ENST00000370503 | SPANXN3 | up | 4.23 | 0.002 |
| ENST00000297375 | EN2 | up | 4.23 | 0.014 |
| ENST00000296871 | CSF2 | up | 4.10 | 0.007 |
| ENST00000265840 | ELMOD1 | up | 4.04 | 0.014 |
| ENST00000252338 | FAM155B | up | 4.03 | 0.005 |
| ENST00000376624 | SPAG6 | up | 3.89 | 0.006 |
| ENST00000569446 | SCRT1 | up | 3.78 | 0.021 |
| ENST00000290974 | ZFYVE28 | up | 3.76 | 0.005 |
| ENST00000461366 | RNF112 | up | 3.72 | 0.000 |
| ENST00000369448 | TENT5C | up | 3.71 | 0.005 |
| ENST00000273550 | FTH1 | up | 3.66 | 0.011 |
| ENST00000429989 | TSPAN14 | up | 3.63 | 0.001 |

**Table S3.** (Continued)

| **Sequence Name** | **Gene Symbol** | **Regulation** | **Fold Change** | **P-value** |
| --- | --- | --- | --- | --- |
| ENST00000273695 | TM4SF19 | up | 3.61 | 0.004 |
| ENST00000297596 | GEM | up | 3.61 | 0.009 |
| ENST00000312562 | FOSL1 | up | 3.58 | 0.007 |
| ENST00000324015 | SERPINF2 | up | 3.56 | 0.007 |
| ENST00000265022 | DGKG | up | 3.51 | 0.003 |
| ENST00000332947 | FAM43B | up | 3.38 | 0.008 |
| ENST00000615198 | CPEB1 | up | 3.33 | 0.006 |
| ENST00000407693 | PTGR1 | up | 3.31 | 0.028 |
| ENST00000458573 | LMOD2 | up | 3.25 | 0.001 |
| ENCT00000264162 | CATG00000054068.1 | up | 3.16 | 0.007 |
| ENST00000264649 | ATP6V0A1 | up | 3.15 | 0.001 |
| ENST00000417669 | MPPED1 | up | 3.12 | 0.036 |
| ENST00000389805 | SQSTM1 | up | 3.10 | 0.004 |
| ENST00000597630 | AC010616.1 | up | 3.06 | 0.018 |
| ENST00000443029 | METTL6 | up | 3.01 | 0.042 |
| ENST00000236877 | SLC8A2 | up | 3.00 | 0.001 |
| ENST00000491143 | ONECUT2 | up | 2.90 | 0.002 |
| ENST00000264808 | PRDM5 | up | 2.84 | 0.030 |
| ENST00000421593 | SLCO1B7 | up | 2.81 | 0.027 |
| ENST00000273861 | SLC10A4 | up | 2.78 | 0.019 |
| ENST00000370597 | CRTAC1 | up | 2.77 | 0.034 |
| ENST00000330055 | ADRA2C | up | 2.74 | 0.006 |
| ENST00000460930 | TCP10 | up | 2.73 | 0.007 |
| ENST00000262041 | MEOX2 | down | 20.77 | 0.003 |
| ENST00000381434 | IL33 | down | 13.08 | 0.013 |
| ENST00000376468 | NPPB | down | 12.56 | 0.001 |
| ENST00000621517 | GJA5 | down | 10.78 | 0.013 |

**Table S3.** (Continued)

| **Sequence Name** | **Gene Symbol** | **Regulation** | **Fold Change** | **P-value** |
| --- | --- | --- | --- | --- |
| ENST00000305817 | PRND | down | 9.70 | 0.024 |
| ENST00000510508 | DIO3 | down | 7.62 | 0.043 |
| ENST00000367609 | ADGRG6 | down | 7.62 | 0.003 |
| ENST00000379747 | POSTN | down | 6.96 | 0.001 |
| ENST00000303924 | HAS2 | down | 6.93 | 0.016 |
| ENST00000295228 | INHBB | down | 6.31 | 0.005 |
| ENST00000618847 | CHST9 | down | 6.29 | 0.003 |
| ENST00000539664 | TRIL | down | 6.26 | 0.018 |
| ENST00000217939 | MXRA5 | down | 6.25 | 0.009 |
| ENST00000409687 | SAPCD2 | down | 6.21 | 0.012 |
| ENST00000256104 | FABP4 | down | 6.19 | 0.002 |
| ENST00000260363 | KIF23 | down | 5.92 | 0.017 |
| ENST00000361228 | RASSF9 | down | 5.90 | 0.000 |
| ENST00000344327 | TRPC6 | down | 5.85 | 0.008 |
| ENST00000296027 | CXCL5 | down | 5.82 | 0.002 |
| ENST00000333425 | PIF1 | down | 5.80 | 0.025 |
| ENST00000428847 | FBXO43 | down | 5.77 | 0.013 |
| ENST00000295666 | IGFBP7 | down | 5.76 | 0.016 |
| ENST00000423059 | THSD7A | down | 5.73 | 0.007 |
| ENST00000327705 | BTNL9 | down | 5.66 | 0.012 |
| ENST00000281961 | TMEM178A | down | 5.51 | 0.003 |
| ENST00000355754 | GBP4 | down | 5.31 | 0.001 |
| ENST0000215980 | CENPM | down | 5.31 | 0.009 |
| ENST00000301691 | SOST | down | 5.30 | 0.015 |
| ENST00000620009 | E2F8 | down | 5.21 | 0.050 |
| ENST00000259486 | ENPP2 | down | 5.13 | 0.030 |
| ENST00000330560 | CDCA2 | down | 5.05 | 0.018 |

**Table S3.** (Continued)

| **Sequence Name** | **Gene Symbol** | **Regulation** | **Fold Change** | **P-value** |
| --- | --- | --- | --- | --- |
| ENST00000282074 | SPC25 | down | 5.00 | 0.036 |
| ENST00000369516 | TSPAN2 | down | 4.86 | 0.005 |
| ENST00000311322 | LPL | down | 4.86 | 0.038 |
| ENST00000244534 | HIST1H1D | down | 4.86 | 0.015 |
| ENST00000329913 | GDF3 | down | 4.73 | 0.039 |
| ENST00000375978 | FOXS1 | down | 4.72 | 0.019 |
| ENST00000289166 | TENT5B | down | 4.71 | 0.012 |
| ENST00000356698 | RSPO3 | down | 4.64 | 0.006 |
| ENST00000402182 | APOBEC3B | down | 4.62 | 0.010 |
| ENST00000284987 | ADAMTS5 | down | 4.58 | 0.002 |
| ENST00000296509 | MAD2L1 | down | 4.55 | 0.026 |
| ENST00000289416 | ACSM3 | down | 4.54 | 0.027 |
| ENST00000373970 | DKK1 | down | 4.54 | 0.018 |
| ENST00000271452 | NUF2 | down | 4.52 | 0.020 |
| ENST00000295927 | PTX3 | down | 4.52 | 0.045 |
| ENST00000316199 | AURKB | down | 4.50 | 0.039 |
| ENST00000559596 | NUSAP1 | down | 4.47 | 0.017 |
| ENST00000263382 | ASF1B | down | 4.47 | 0.046 |
| ENST00000359062 | PDE3A | down | 4.46 | 0.006 |

**Table S4.** mRNA-lncRNA pairs identified in the expression network for upregulated lncRNAs.

| **mRNA** | **LncRNA** | **PCC** | **Interaction** | **P-value** |
| --- | --- | --- | --- | --- |
| ADAM22 | AC004988.1 | 0.955197 | + | 0.000217 |
| ADRA2C | AC004988.1 | 0.950113 | + | 0.000299 |
| AEBP2 | AC004988.1 | -0.95739 | - | 0.000187 |
| ANKRD13B | AC004988.1 | 0.960633 | + | 0.000148 |
| ATP6V0D2 | AC004988.1 | 0.974675 | + | 3.98E-05 |
| ATP6V1FNB | AC004988.1 | 0.958163 | + | 0.000177 |
| BATF | AC004988.1 | 0.950882 | + | 0.000285 |
| BEST1 | AC004988.1 | 0.978092 | + | 2.59E-05 |
| C17orf67 | AC004988.1 | -0.96192 | - | 0.000134 |
| C22orf15 | AC004988.1 | -0.95029 | - | 0.000296 |
| CATG00000002338.1 | AC004988.1 | -0.967 | - | 8.76E-05 |
| CBX5 | AC004988.1 | -0.95239 | - | 0.00026 |
| CCDC142 | AC004988.1 | 0.969514 | + | 6.92E-05 |
| CD22 | AC004988.1 | 0.980237 | + | 1.90E-05 |
| CEP57L1 | AC004988.1 | -0.95037 | - | 0.000294 |
| CLEC16A | AC004988.1 | 0.961263 | + | 0.000141 |
| CXCL16 | AC004988.1 | 0.984027 | + | 1.01E-05 |
| DLX1 | AC004988.1 | -0.95181 | - | 0.00027 |
| EARS2 | AC004988.1 | -0.97373 | - | 4.44E-05 |
| ELOA2 | AC004988.1 | 0.962486 | + | 0.000128 |
| ENY2 | AC004988.1 | -0.97261 | - | 5.03E-05 |
| FAM155B | AC004988.1 | 0.961168 | + | 0.000142 |
| FTL | AC004988.1 | 0.970807 | + | 6.08E-05 |
| FXR2 | AC004988.1 | 0.98305 | + | 1.20E-05 |
| GLS | AC004988.1 | 0.957919 | + | 0.00018 |
| GPC6 | AC004988.1 | -0.96514 | - | 0.000103 |
| GPR85 | AC004988.1 | -0.97409 | - | 4.26E-05 |

**Table S4.** (Continued)

| **mRNA** | **LncRNA** | **PCC** | **Interaction** | **P-value** |
| --- | --- | --- | --- | --- |
| GUCY1A2 | AC004988.1 | -0.98523 | - | 7.97E-06 |
| HIST1H1D | AC004988.1 | -0.96252 | - | 0.000128 |
| HNRNPA0 | AC004988.1 | -0.95281 | - | 0.000253 |
| HUS1B | AC004988.1 | 0.976048 | + | 3.37E-05 |
| IER3IP1 | AC004988.1 | -0.95207 | - | 0.000266 |
| INA | AC004988.1 | 0.962671 | + | 0.000126 |
| ISOC2 | AC004988.1 | -0.95286 | - | 0.000253 |
| KIAA1658 | AC004988.1 | 0.962367 | + | 0.00013 |
| LCTL | AC004988.1 | -0.976 | - | 3.39E-05 |
| LSM4 | AC004988.1 | -0.97723 | - | 2.90E-05 |
| MEGF9 | AC004988.1 | -0.96956 | - | 6.89E-05 |
| MRGPRG | AC004988.1 | 0.972418 | + | 5.14E-05 |
| MXRA5 | AC004988.1 | -0.97726 | - | 2.89E-05 |
| NSFL1C | AC004988.1 | -0.95067 | - | 0.000289 |
| NSMCE3 | AC004988.1 | -0.96308 | - | 0.000122 |
| PDAP1 | AC004988.1 | -0.96086 | - | 0.000145 |
| PIGS | AC004988.1 | 0.960731 | + | 0.000147 |
| PRR29 | AC004988.1 | 0.957524 | + | 0.000186 |
| PYCR1 | AC004988.1 | -0.95457 | - | 0.000227 |
| RRAGD | AC004988.1 | 0.97037 | + | 6.36E-05 |
| RSPO3 | AC004988.1 | -0.962 | - | 0.000133 |
| SERPINF2 | AC004988.1 | 0.979562 | + | 2.10E-05 |
| SLC7A11 | AC004988.1 | 0.963641 | + | 0.000117 |
| STAT3 | AC004988.1 | 0.95061 | + | 0.00029 |
| TFEC | AC004988.1 | 0.960064 | + | 0.000155 |
| THSD7A | AC004988.1 | -0.96589 | - | 9.67E-05 |
| TMEM107 | AC004988.1 | 0.952946 | + | 0.000251 |

**Table S4.** (Continued)

| **mRNA** | **LncRNA** | **PCC** | **Interaction** | **P-value** |
| --- | --- | --- | --- | --- |
| TMEM178A | AC004988.1 | -0.95049 | - | 0.000292 |
| TMEM87B | AC004988.1 | 0.974751 | + | 3.95E-05 |
| TMX4 | AC004988.1 | 0.974661 | + | 3.99E-05 |
| TRIL | AC004988.1 | -0.96329 | - | 0.00012 |
| TRPC6 | AC004988.1 | -0.97981 | - | 2.03E-05 |
| TSPAN14 | AC004988.1 | 0.953538 | + | 0.000242 |
| TTC23L | AC004988.1 | -0.95099 | - | 0.000284 |
| UCP2 | AC004988.1 | -0.9537 | - | 0.00024 |
| VPS18 | AC004988.1 | 0.965274 | + | 0.000102 |
| ZNF148 | AC004988.1 | -0.96139 | - | 0.00014 |
| ZNF792 | AC004988.1 | 0.969571 | + | 6.88E-05 |
| CYP1B1 | CYP1B1 | 0.994926 | + | 3.25E-07 |
| LTBP4 | CYP1B1 | -0.96832 | - | 7.76E-05 |
| LY96 | CYP1B1 | 0.965573 | + | 9.94E-05 |
| MCOLN1 | CYP1B1 | 0.959258 | + | 0.000164 |
| NCAPH | CYP1B1 | 0.961335 | + | 0.00014 |
| RASSF7 | CYP1B1 | 0.974098 | + | 4.26E-05 |
| AKAP8L | LUCAT1 | 0.96639 | + | 9.25E-05 |
| BCL6 | LUCAT1 | 0.976727 | + | 3.10E-05 |
| C2orf81 | LUCAT1 | 0.978316 | + | 2.51E-05 |
| CDYL2 | LUCAT1 | 0.976336 | + | 3.25E-05 |
| CUEDC1 | LUCAT1 | 0.988087 | + | 4.19E-06 |
| CYSTM1 | LUCAT1 | 0.954424 | + | 0.000229 |
| DOCK11 | LUCAT1 | 0.967125 | + | 8.67E-05 |
| EN2 | LUCAT1 | 0.968531 | + | 7.61E-05 |
| FAM107B | LUCAT1 | -0.95174 | - | 0.000271 |
| FLCN | LUCAT1 | 0.974355 | + | 4.14E-05 |

**Table S4.** (Continued)

| **mRNA** | **LncRNA** | **PCC** | **Interaction** | **P-value** |
| --- | --- | --- | --- | --- |
| FNIP1 | LUCAT1 | 0.955939 | + | 0.000207 |
| FTH1 | LUCAT1 | 0.953328 | + | 0.000245 |
| HSBP1L1 | LUCAT1 | 0.956277 | + | 0.000202 |
| IL18R1 | LUCAT1 | 0.966898 | + | 8.84E-05 |
| IQCG | LUCAT1 | 0.974645 | + | 4.00E-05 |
| KCNJ15 | LUCAT1 | 0.971427 | + | 5.71E-05 |
| KCNQ1 | LUCAT1 | 0.970898 | + | 6.03E-05 |
| MASP1 | LUCAT1 | -0.97538 | - | 3.66E-05 |
| MCOLN1 | LUCAT1 | 0.968292 | + | 7.78E-05 |
| PGD | LUCAT1 | 0.979654 | + | 2.07E-05 |
| PPP1R15A | LUCAT1 | 0.958859 | + | 0.000169 |
| PTGR1 | LUCAT1 | 0.96364 | + | 0.000117 |
| RDH10 | LUCAT1 | 0.981354 | + | 1.60E-05 |
| SLC6A6 | LUCAT1 | 0.975128 | + | 3.78E-05 |
| SQSTM1 | LUCAT1 | 0.977554 | + | 2.78E-05 |
| STC1 | LUCAT1 | 0.950759 | + | 0.000288 |
| STX1A | LUCAT1 | 0.958887 | + | 0.000168 |
| TBATA | LUCAT1 | -0.95382 | - | 0.000238 |
| TCP10 | LUCAT1 | 0.964039 | + | 0.000113 |
| TGFB2 | LUCAT1 | -0.98415 | - | 9.84E-06 |
| TOM1 | LUCAT1 | 0.959951 | + | 0.000156 |
| TRPC4 | LUCAT1 | -0.95769 | - | 0.000183 |
| TSPAN18 | LUCAT1 | -0.95806 | - | 0.000179 |
| UNC5CL | LUCAT1 | 0.966514 | + | 9.15E-05 |
| WBP1 | LUCAT1 | 0.977264 | + | 2.89E-05 |
| ANKRD13B | TFEC | 0.973293 | + | 4.67E-05 |
| ATP6V0D2 | TFEC | 0.965524 | + | 9.98E-05 |

**Table S4.** (Continued)

| **mRNA** | **LncRNA** | **PCC** | **Interaction** | **P-value** |
| --- | --- | --- | --- | --- |
| CATG00000010950.1 | TFEC | -0.95463 | - | 0.000226 |
| CATG00000031483.1 | TFEC | -0.96125 | - | 0.000141 |
| CATG00000107419.1 | TFEC | -0.95994 | - | 0.000156 |
| CHPF2 | TFEC | 0.970445 | + | 6.31E-05 |
| DOCK11 | TFEC | 0.959254 | + | 0.000164 |
| FNIP1 | TFEC | 0.953526 | + | 0.000242 |
| FOSL1 | TFEC | 0.955749 | + | 0.000209 |
| FTH1 | TFEC | 0.966814 | + | 8.91E-05 |
| FTL | TFEC | 0.958222 | + | 0.000177 |
| GLS | TFEC | 0.952082 | + | 0.000265 |
| GPR85 | TFEC | -0.95768 | - | 0.000183 |
| IL18R1 | TFEC | 0.95655 | + | 0.000198 |
| KHDRBS2 | TFEC | 0.953179 | + | 0.000248 |
| KIAA1658 | TFEC | 0.95142 | + | 0.000276 |
| LCTL | TFEC | -0.97402 | - | 4.30E-05 |
| LSM4 | TFEC | -0.97047 | - | 6.30E-05 |
| LY96 | TFEC | 0.983587 | + | 1.09E-05 |
| MCOLN1 | TFEC | 0.963702 | + | 0.000116 |
| NARS2 | TFEC | -0.95269 | - | 0.000256 |
| NSMCE3 | TFEC | -0.96798 | - | 8.01E-05 |
| RASSF7 | TFEC | 0.971317 | + | 5.77E-05 |
| SH3KBP1 | TFEC | 0.96114 | + | 0.000142 |
| SLC7A11 | TFEC | 0.992937 | + | 8.76E-07 |
| SPANXN3 | TFEC | 0.964618 | + | 0.000108 |
| SQSTM1 | TFEC | 0.974026 | + | 4.30E-05 |
| SSR3 | TFEC | 0.957131 | + | 0.000191 |
| TFEC | TFEC | 0.995512 | + | 2.25E-07 |

**Table S4.** (Continued)

| **mRNA** | **LncRNA** | **PCC** | **Interaction** | **P-value** |
| --- | --- | --- | --- | --- |
| CATG00000010950.1 | TFEC | -0.95463 | - | 0.000226 |
| CATG00000031483.1 | TFEC | -0.96125 | - | 0.000141 |
| CATG00000107419.1 | TFEC | -0.95994 | - | 0.000156 |
| CHPF2 | TFEC | 0.970445 | + | 6.31E-05 |
| DOCK11 | TFEC | 0.959254 | + | 0.000164 |
| FNIP1 | TFEC | 0.953526 | + | 0.000242 |
| FOSL1 | TFEC | 0.955749 | + | 0.000209 |
| FTH1 | TFEC | 0.966814 | + | 8.91E-05 |
| FTL | TFEC | 0.958222 | + | 0.000177 |
| GLS | TFEC | 0.952082 | + | 0.000265 |
| GPR85 | TFEC | -0.95768 | - | 0.000183 |
| IL18R1 | TFEC | 0.95655 | + | 0.000198 |
| KHDRBS2 | TFEC | 0.953179 | + | 0.000248 |
| KIAA1658 | TFEC | 0.95142 | + | 0.000276 |
| LCTL | TFEC | -0.97402 | - | 4.30E-05 |
| LSM4 | TFEC | -0.97047 | - | 6.30E-05 |
| LY96 | TFEC | 0.983587 | + | 1.09E-05 |
| MCOLN1 | TFEC | 0.963702 | + | 0.000116 |
| NARS2 | TFEC | -0.95269 | - | 0.000256 |
| NSMCE3 | TFEC | -0.96798 | - | 8.01E-05 |
| RASSF7 | TFEC | 0.971317 | + | 5.77E-05 |
| SH3KBP1 | TFEC | 0.96114 | + | 0.000142 |
| SLC7A11 | TFEC | 0.992937 | + | 8.76E-07 |
| SPANXN3 | TFEC | 0.964618 | + | 0.000108 |
| SQSTM1 | TFEC | 0.974026 | + | 4.30E-05 |
| SSR3 | TFEC | 0.957131 | + | 0.000191 |
| TFEC | TFEC | 0.995512 | + | 2.25E-07 |

**Table S4.** (Continued)

| **mRNA** | **LncRNA** | **PCC** | **Interaction** | **P-value** |
| --- | --- | --- | --- | --- |
| TGFB2 | TFEC | -0.97956 | - | 2.10E-05 |
| TMEM107 | TFEC | 0.961408 | + | 0.00014 |
| TMEM87B | TFEC | 0.954009 | + | 0.000235 |
| TOM1 | TFEC | 0.960635 | + | 0.000148 |
| WBP1 | TFEC | 0.954992 | + | 0.00022 |
| XKR8 | TFEC | 0.95939 | + | 0.000162 |
| C22orf15 | AC108676.1 | -0.95014 | - | 0.000298 |
| CATG00000034215.1 | AC108676.1 | -0.9708 | - | 6.09E-05 |
| CSF2 | AC108676.1 | 0.951563 | + | 0.000274 |
| DCLRE1C | AC108676.1 | -0.95109 | - | 0.000282 |
| DUSP9 | AC108676.1 | 0.952924 | + | 0.000252 |
| ERN1 | AC108676.1 | 0.995025 | + | 3.07E-07 |
| LMCD1 | AC108676.1 | -0.95397 | - | 0.000235 |
| MSH5-SAPCD1 | AC108676.1 | -0.95132 | - | 0.000278 |
| NCKAP5 | AC108676.1 | -0.9802 | - | 1.91E-05 |
| PHACTR2 | AC108676.1 | -0.9843 | - | 9.57E-06 |
| RAB9A | AC108676.1 | 0.952554 | + | 0.000258 |
| RNF112 | AC108676.1 | 0.978926 | + | 2.30E-05 |
| SLC41A2 | AC108676.1 | 0.969367 | + | 7.02E-05 |
| THBD | AC108676.1 | -0.95022 | - | 0.000297 |
| TOMM7 | AC108676.1 | -0.95525 | - | 0.000217 |
| UCN | AC108676.1 | 0.951787 | + | 0.00027 |

**Table S5.** mRNA-lncRNA pairs identified in the expression network for downregulated lncRNAs.

| **mRNA** | **LncRNA** | **PCC** | **Interaction** | **P-value** |
| --- | --- | --- | --- | --- |
| ACSM2A | CATG00000097867.1 | 0.993137 | + | 8.04E-07 |
| ADGRG6 | CATG00000097867.1 | 0.976282 | + | 3.28E-05 |
| AP3S1 | CATG00000097867.1 | -0.9818 | - | 1.49E-05 |
| CATG00000002338.1 | CATG00000097867.1 | 0.967259 | + | 8.56E-05 |
| CATG00000013249.1 | CATG00000097867.1 | 0.967387 | + | 8.46E-05 |
| COX4I1 | CATG00000097867.1 | 0.953396 | + | 0.000244 |
| EARS2 | CATG00000097867.1 | 0.954119 | + | 0.000233 |
| FBLIM1 | CATG00000097867.1 | 0.976074 | + | 3.36E-05 |
| FOSL1 | CATG00000097867.1 | -0.96906 | - | 7.23E-05 |
| GLIPR2 | CATG00000097867.1 | 0.980003 | + | 1.97E-05 |
| GPC6 | CATG00000097867.1 | 0.977371 | + | 2.85E-05 |
| GPR85 | CATG00000097867.1 | 0.953762 | + | 0.000239 |
| IGFBP5 | CATG00000097867.1 | 0.957993 | + | 0.00018 |
| LRRC17 | CATG00000097867.1 | 0.974811 | + | 3.92E-05 |
| PCSK1 | CATG00000097867.1 | 0.956186 | + | 0.000203 |
| PDK2 | CATG00000097867.1 | 0.961802 | + | 0.000135 |
| TRIL | CATG00000097867.1 | 0.95833 | + | 0.000175 |
| ZKSCAN1 | CATG00000097867.1 | -0.95132 | - | 0.000278 |
| AC037459.1 | AL031710.2 | 0.970929 | + | 6.01E-05 |
| AC087645.1 | AL031710.2 | 0.967073 | + | 8.71E-05 |
| AC092329.3 | AL031710.2 | 0.952795 | + | 0.000254 |
| ACADS | AL031710.2 | 0.953061 | + | 0.00025 |
| ALDH7A1 | AL031710.2 | 0.959972 | + | 0.000156 |
| ANLN | AL031710.2 | 0.964666 | + | 0.000107 |
| APOBEC3C | AL031710.2 | 0.958341 | + | 0.000175 |
| ARHGAP11A | AL031710.2 | 0.957588 | + | 0.000185 |
| ASPM | AL031710.2 | 0.966294 | + | 9.33E-05 |

**Table S5.** (Continued)

| **mRNA** | **LncRNA** | **PCC** | **Interaction** | **P-value** |
| --- | --- | --- | --- | --- |
| ATPAF1 | AL031710.2 | 0.953669 | + | 0.00024 |
| AURKA | AL031710.2 | 0.983209 | + | 1.17E-05 |
| AURKB | AL031710.2 | 0.965377 | + | 0.000101 |
| BRCA2 | AL031710.2 | 0.971429 | + | 5.71E-05 |
| CATG00000063141.1 | AL031710.2 | -0.96337 | - | 0.00012 |
| CCM2L | AL031710.2 | -0.95021 | - | 0.000297 |
| CCNA2 | AL031710.2 | 0.957077 | + | 0.000191 |
| CCNB1 | AL031710.2 | 0.95399 | + | 0.000235 |
| CD22 | AL031710.2 | -0.96523 | - | 0.000102 |
| CDC25C | AL031710.2 | 0.975566 | + | 3.58E-05 |
| CDCA2 | AL031710.2 | 0.961151 | + | 0.000142 |
| CDKN3 | AL031710.2 | 0.958407 | + | 0.000174 |
| CDT1 | AL031710.2 | 0.967882 | + | 8.08E-05 |
| CENPE | AL031710.2 | 0.960796 | + | 0.000146 |
| CENPM | AL031710.2 | 0.974575 | + | 4.03E-05 |
| CEP57L1 | AL031710.2 | 0.975065 | + | 3.80E-05 |
| CEP72 | AL031710.2 | 0.957231 | + | 0.000189 |
| CHAF1B | AL031710.2 | 0.963212 | + | 0.000121 |
| CHTF18 | AL031710.2 | 0.96813 | + | 7.90E-05 |
| DEPDC1 | AL031710.2 | 0.966121 | + | 9.48E-05 |
| DEPDC1B | AL031710.2 | 0.973189 | + | 4.72E-05 |
| DHODH | AL031710.2 | 0.972696 | + | 4.99E-05 |
| DIAPH3 | AL031710.2 | 0.952912 | + | 0.000252 |
| DLGAP5 | AL031710.2 | 0.956877 | + | 0.000194 |
| DTYMK | AL031710.2 | 0.96123 | + | 0.000141 |
| EIF3M | AL031710.2 | 0.95089 | + | 0.000285 |
| EVPLL | AL031710.2 | -0.95767 | - | 0.000184 |

**Table S5.** (Continued)

| **mRNA** | **LncRNA** | **PCC** | **Interaction** | **P-value** |
| --- | --- | --- | --- | --- |
| FABP4 | AL031710.2 | 0.984079 | + | 9.97E-06 |
| FAM110D | AL031710.2 | 0.96814 | + | 7.89E-05 |
| FAT4 | AL031710.2 | 0.96302 | + | 0.000123 |
| FBXL2 | AL031710.2 | 0.954333 | + | 0.00023 |
| FBXO43 | AL031710.2 | 0.985269 | + | 7.90E-06 |
| FIP1L1 | AL031710.2 | -0.95135 | - | 0.000277 |
| FOXS1 | AL031710.2 | 0.96287 | + | 0.000124 |
| GEN1 | AL031710.2 | 0.962341 | + | 0.00013 |
| GIMAP7 | AL031710.2 | 0.962157 | + | 0.000132 |
| GINS1 | AL031710.2 | 0.978317 | + | 2.51E-05 |
| GINS2 | AL031710.2 | 0.961162 | + | 0.000142 |
| GSPT1 | AL031710.2 | 0.961698 | + | 0.000136 |
| GSTCD | AL031710.2 | 0.964799 | + | 0.000106 |
| GTSE1 | AL031710.2 | 0.959395 | + | 0.000162 |
| HIST1H1D | AL031710.2 | 0.955345 | + | 0.000215 |
| HIST1H2BI | AL031710.2 | 0.95452 | + | 0.000227 |
| HIST1H4A | AL031710.2 | 0.966055 | + | 9.53E-05 |
| HIST1H4J | AL031710.2 | 0.951258 | + | 0.000279 |
| HNRNPA0 | AL031710.2 | 0.961668 | + | 0.000137 |
| HSPG2 | AL031710.2 | 0.970705 | + | 6.15E-05 |
| JMJD7-PLA2G4B | AL031710.2 | 0.962637 | + | 0.000127 |
| KIF18A | AL031710.2 | 0.955527 | + | 0.000213 |
| KIF23 | AL031710.2 | 0.974699 | + | 3.97E-05 |
| KIF4B | AL031710.2 | 0.953421 | + | 0.000244 |
| KIFC1 | AL031710.2 | 0.972824 | + | 4.92E-05 |
| KPNB1 | AL031710.2 | 0.950566 | + | 0.000291 |
| MAD2L1 | AL031710.2 | 0.978565 | + | 2.42E-05 |

**Table S5.** (Continued)

| **mRNA** | **LncRNA** | **PCC** | **Interaction** | **P-value** |
| --- | --- | --- | --- | --- |
| MAP2K6 | AL031710.2 | 0.959464 | + | 0.000161 |
| MED17 | AL031710.2 | 0.962457 | + | 0.000129 |
| MEGF9 | AL031710.2 | 0.969635 | + | 6.84E-05 |
| MOCS3 | AL031710.2 | 0.964863 | + | 0.000106 |
| MPZL1 | AL031710.2 | 0.96488 | + | 0.000105 |
| MRGPRG | AL031710.2 | -0.95181 | - | 0.00027 |
| MTR | AL031710.2 | 0.960878 | + | 0.000145 |
| MYEF2 | AL031710.2 | 0.964026 | + | 0.000113 |
| NCAPG | AL031710.2 | 0.955435 | + | 0.000214 |
| NRIP1 | AL031710.2 | 0.967856 | + | 8.10E-05 |
| NUF2 | AL031710.2 | 0.973216 | + | 4.71E-05 |
| NUS1 | AL031710.2 | 0.968946 | + | 7.31E-05 |
| NUSAP1 | AL031710.2 | 0.970526 | + | 6.26E-05 |
| NXPH2 | AL031710.2 | 0.968458 | + | 7.66E-05 |
| OOEP | AL031710.2 | -0.96799 | - | 8.00E-05 |
| OPN1SW | AL031710.2 | 0.979659 | + | 2.07E-05 |
| PCSK6 | AL031710.2 | -0.97274 | - | 4.96E-05 |
| PDAP1 | AL031710.2 | 0.96847 | + | 7.65E-05 |
| PF4 | AL031710.2 | 0.953792 | + | 0.000238 |
| PIF1 | AL031710.2 | 0.960884 | + | 0.000145 |
| PKN3 | AL031710.2 | 0.956438 | + | 0.0002 |
| PLK1 | AL031710.2 | 0.950356 | + | 0.000295 |
| PML | AL031710.2 | 0.962145 | + | 0.000132 |
| POLA2 | AL031710.2 | 0.952957 | + | 0.000251 |
| POLE2 | AL031710.2 | 0.95146 | + | 0.000276 |
| PSPC1 | AL031710.2 | 0.969477 | + | 6.95E-05 |
| PYROXD2 | AL031710.2 | 0.971067 | + | 5.92E-05 |

**Table S5.** (Continued)

| **mRNA** | **LncRNA** | **PCC** | **Interaction** | **P-value** |
| --- | --- | --- | --- | --- |
| RNASE13 | AL031710.2 | -0.95358 | - | 0.000241 |
| RPL39L | AL031710.2 | 0.961742 | + | 0.000136 |
| RRM1 | AL031710.2 | 0.973722 | + | 4.45E-05 |
| RYBP | AL031710.2 | 0.964853 | + | 0.000106 |
| SAAL1 | AL031710.2 | 0.95477 | + | 0.000224 |
| SAPCD2 | AL031710.2 | 0.97236 | + | 5.17E-05 |
| SAR1A | AL031710.2 | 0.955879 | + | 0.000208 |
| SELENOS | AL031710.2 | 0.96549 | + | 0.0001 |
| SERINC5 | AL031710.2 | 0.95314 | + | 0.000248 |
| SKP1 | AL031710.2 | 0.956228 | + | 0.000203 |
| SLC8A2 | AL031710.2 | -0.965 | - | 0.000104 |
| SNRPE | AL031710.2 | 0.959816 | + | 0.000157 |
| SPC24 | AL031710.2 | 0.984806 | + | 8.67E-06 |
| SPC25 | AL031710.2 | 0.969647 | + | 6.83E-05 |
| SRPK1 | AL031710.2 | 0.954458 | + | 0.000228 |
| THOP1 | AL031710.2 | 0.977349 | + | 2.86E-05 |
| TMEM231 | AL031710.2 | 0.952732 | + | 0.000255 |
| TMEM237 | AL031710.2 | 0.953659 | + | 0.00024 |
| TOP2A | AL031710.2 | 0.953922 | + | 0.000236 |
| TRAIP | AL031710.2 | 0.972473 | + | 5.11E-05 |
| TRIP13 | AL031710.2 | 0.954435 | + | 0.000228 |
| TTC23L | AL031710.2 | 0.971309 | + | 5.78E-05 |
| TTC39C | AL031710.2 | -0.95447 | - | 0.000228 |
| TTK | AL031710.2 | 0.958272 | + | 0.000176 |
| TYMS | AL031710.2 | 0.958734 | + | 0.00017 |
| UBE2C | AL031710.2 | 0.950083 | + | 0.000299 |
| UQCR10 | AL031710.2 | 0.971621 | + | 5.59E-05 |

**Table S5.** (Continued)

| **mRNA** | **LncRNA** | **PCC** | **Interaction** | **P-value** |
| --- | --- | --- | --- | --- |
| ZNF641 | AL031710.2 | 0.969023 | + | 7.26E-05 |
| ZWINT | AL031710.2 | 0.965064 | + | 0.000104 |
| AC119673.1 | TNFSF10 | 0.955843 | + | 0.000208 |
| AEBP2 | TNFSF10 | 0.963826 | + | 0.000115 |
| CALHM5 | TNFSF10 | 0.968255 | + | 7.81E-05 |
| CATG00000002338.1 | TNFSF10 | 0.951956 | + | 0.000267 |
| CBX5 | TNFSF10 | 0.985247 | + | 7.94E-06 |
| CD22 | TNFSF10 | -0.95986 | - | 0.000157 |
| CDCA2 | TNFSF10 | 0.956138 | + | 0.000204 |
| CEP57L1 | TNFSF10 | 0.976086 | + | 3.36E-05 |
| CXCL16 | TNFSF10 | -0.96982 | - | 6.72E-05 |
| CYLD | TNFSF10 | 0.964859 | + | 0.000106 |
| DCTPP1 | TNFSF10 | 0.963792 | + | 0.000115 |
| DOLPP1 | TNFSF10 | -0.97585 | - | 3.46E-05 |
| EARS2 | TNFSF10 | 0.963115 | + | 0.000122 |
| ELOA2 | TNFSF10 | -0.9648 | - | 0.000106 |
| ENY2 | TNFSF10 | 0.98943 | + | 2.93E-06 |
| FANCL | TNFSF10 | 0.951676 | + | 0.000272 |
| FSD2 | TNFSF10 | -0.96382 | - | 0.000115 |
| FTL | TNFSF10 | -0.952 | - | 0.000267 |
| FXR2 | TNFSF10 | -0.96356 | - | 0.000118 |
| GEN1 | TNFSF10 | 0.956083 | + | 0.000205 |
| GJA5 | TNFSF10 | 0.955819 | + | 0.000209 |
| GPC6 | TNFSF10 | 0.962067 | + | 0.000133 |
| GPR85 | TNFSF10 | 0.950086 | + | 0.000299 |
| HELLS | TNFSF10 | 0.973786 | + | 4.42E-05 |
| HIST1H1D | TNFSF10 | 0.964902 | + | 0.000105 |

**Table S5.** (Continued)

| **mRNA** | **LncRNA** | **PCC** | **Interaction** | **P-value** |
| --- | --- | --- | --- | --- |
| HNRNPA0 | TNFSF10 | 0.976106 | + | 3.35E-05 |
| HSPG2 | TNFSF10 | 0.963929 | + | 0.000114 |
| HUS1B | TNFSF10 | -0.95032 | - | 0.000295 |
| INA | TNFSF10 | -0.96502 | - | 0.000104 |
| ISOC2 | TNFSF10 | 0.957334 | + | 0.000188 |
| LCTL | TNFSF10 | 0.957503 | + | 0.000186 |
| LSM4 | TNFSF10 | 0.957675 | + | 0.000184 |
| MEGF9 | TNFSF10 | 0.975347 | + | 3.68E-05 |
| MXRA5 | TNFSF10 | 0.96148 | + | 0.000139 |
| NDUFB8 | TNFSF10 | 0.957617 | + | 0.000184 |
| PCSK6 | TNFSF10 | -0.95499 | - | 0.00022 |
| PIGS | TNFSF10 | -0.97467 | - | 3.98E-05 |
| PRR29 | TNFSF10 | -0.95311 | - | 0.000249 |
| PYCR1 | TNFSF10 | 0.96886 | + | 7.37E-05 |
| RASSF9 | TNFSF10 | 0.958499 | + | 0.000173 |
| RSPO3 | TNFSF10 | 0.981346 | + | 1.60E-05 |
| SERPINF2 | TNFSF10 | -0.97864 | - | 2.40E-05 |
| SIGLEC1 | TNFSF10 | 0.951711 | + | 0.000271 |
| TCF4 | TNFSF10 | -0.95566 | - | 0.000211 |
| TENT5B | TNFSF10 | 0.960602 | + | 0.000148 |
| TEX13A | TNFSF10 | 0.951048 | + | 0.000283 |
| THSD7A | TNFSF10 | 0.980965 | + | 1.70E-05 |
| TRIL | TNFSF10 | 0.965547 | + | 9.96E-05 |
| TRPC6 | TNFSF10 | 0.971307 | + | 5.78E-05 |
| TSPAN2 | TNFSF10 | 0.972438 | + | 5.13E-05 |
| USP31 | TNFSF10 | -0.95381 | - | 0.000238 |
| WDR70 | TNFSF10 | 0.95582 | + | 0.000208 |

**Table S5.** (Continued)

| **mRNA** | **LncRNA** | **PCC** | **Interaction** | **P-value** |
| --- | --- | --- | --- | --- |
| ACSM2A | ACSM3 | 0.970141 | + | 6.51E-05 |
| ADCYAP1 | ACSM3 | -0.95347 | - | 0.000243 |
| ADGRG6 | ACSM3 | 0.971233 | + | 5.82E-05 |
| AP3S1 | ACSM3 | -0.96483 | - | 0.000106 |
| BMPER | ACSM3 | 0.95697 | + | 0.000193 |
| CATG00000013249.1 | ACSM3 | 0.984321 | + | 9.52E-06 |
| CCIN | ACSM3 | 0.969639 | + | 6.84E-05 |
| CHST2 | ACSM3 | 0.955358 | + | 0.000215 |
| EFNB2 | ACSM3 | 0.968994 | + | 7.28E-05 |
| ELOA2 | ACSM3 | -0.9529 | - | 0.000252 |
| FAM160B1 | ACSM3 | -0.95215 | - | 0.000264 |
| FAM27D1 | ACSM3 | 0.955959 | + | 0.000207 |
| FBLIM1 | ACSM3 | 0.964575 | + | 0.000108 |
| FHL1 | ACSM3 | 0.955017 | + | 0.00022 |
| FNIP1 | ACSM3 | -0.95014 | - | 0.000298 |
| FOSL1 | ACSM3 | -0.97052 | - | 6.27E-05 |
| GLIPR2 | ACSM3 | 0.969488 | + | 6.94E-05 |
| GPC6 | ACSM3 | 0.951732 | + | 0.000271 |
| IL10RA | ACSM3 | 0.95845 | + | 0.000174 |
| LCE3D | ACSM3 | -0.95246 | - | 0.000259 |
| MYL9 | ACSM3 | 0.968391 | + | 7.71E-05 |
| ORMDL3 | ACSM3 | -0.954 | - | 0.000235 |
| PEG10 | ACSM3 | 0.968189 | + | 7.86E-05 |
| PPP2R2A | ACSM3 | 0.961827 | + | 0.000135 |
| SETBP1 | ACSM3 | 0.954006 | + | 0.000235 |
| SFMBT1 | ACSM3 | -0.96381 | - | 0.000115 |
| USP31 | ACSM3 | -0.97214 | - | 5.29E-05 |

**Table S5.** (Continued)

| **mRNA** | **LncRNA** | **PCC** | **Interaction** | **P-value** |
| --- | --- | --- | --- | --- |
| ABHD17B | AC093510.2 | 0.973821 | + | 4.40E-05 |
| AC037459.1 | AC093510.2 | 0.961407 | + | 0.00014 |
| ADAM22 | AC093510.2 | -0.95835 | - | 0.000175 |
| AEBP2 | AC093510.2 | 0.974508 | + | 4.06E-05 |
| ALDH7A1 | AC093510.2 | 0.969743 | + | 6.77E-05 |
| ANXA1 | AC093510.2 | 0.956761 | + | 0.000196 |
| APOBEC3C | AC093510.2 | 0.973318 | + | 4.65E-05 |
| ARL3 | AC093510.2 | 0.951569 | + | 0.000274 |
| ATP8B1 | AC093510.2 | 0.950405 | + | 0.000294 |
| ATPAF1 | AC093510.2 | 0.957343 | + | 0.000188 |
| BCLAF1 | AC093510.2 | 0.980336 | + | 1.87E-05 |
| BMP7 | AC093510.2 | -0.96047 | - | 0.00015 |
| C17orf67 | AC093510.2 | 0.974578 | + | 4.03E-05 |
| C22orf15 | AC093510.2 | 0.960687 | + | 0.000147 |
| C22orf39 | AC093510.2 | 0.981688 | + | 1.51E-05 |
| CALHM5 | AC093510.2 | 0.965736 | + | 9.80E-05 |
| CALM3 | AC093510.2 | 0.95518 | + | 0.000218 |
| CATG00000075035.1 | AC093510.2 | -0.97453 | - | 4.05E-05 |
| CATG00000086309.1 | AC093510.2 | 0.97609 | + | 3.36E-05 |
| CCDC142 | AC093510.2 | -0.97252 | - | 5.08E-05 |
| CCDC88B | AC093510.2 | 0.959193 | + | 0.000165 |
| CD22 | AC093510.2 | -0.97628 | - | 3.28E-05 |
| CD48 | AC093510.2 | -0.95939 | - | 0.000162 |
| CEP57L1 | AC093510.2 | 0.95077 | + | 0.000287 |
| CIZ1 | AC093510.2 | 0.981908 | + | 1.46E-05 |
| CLDN7 | AC093510.2 | 0.963745 | + | 0.000116 |
| CMTR2 | AC093510.2 | 0.971149 | + | 5.87E-05 |

**Table S5.** (Continued)

| **mRNA** | **LncRNA** | **PCC** | **Interaction** | **P-value** |
| --- | --- | --- | --- | --- |
| CNOT7 | AC093510.2 | 0.961821 | + | 0.000135 |
| COPS8 | AC093510.2 | 0.953074 | + | 0.000249 |
| CRIM1 | AC093510.2 | 0.975411 | + | 3.65E-05 |
| DBNL | AC093510.2 | 0.971448 | + | 5.70E-05 |
| DEPDC1B | AC093510.2 | 0.957859 | + | 0.000181 |
| DGKG | AC093510.2 | -0.97331 | - | 4.66E-05 |
| DGKH | AC093510.2 | 0.953983 | + | 0.000235 |
| DLX1 | AC093510.2 | 0.985908 | + | 6.92E-06 |
| DSC2 | AC093510.2 | 0.958808 | + | 0.000169 |
| E2F8 | AC093510.2 | 0.960972 | + | 0.000144 |
| EIF1 | AC093510.2 | 0.968632 | + | 7.54E-05 |
| EIF3M | AC093510.2 | 0.954014 | + | 0.000235 |
| ELSPBP1 | AC093510.2 | -0.95854 | - | 0.000173 |
| EPSTI1 | AC093510.2 | 0.969102 | + | 7.20E-05 |
| FABP4 | AC093510.2 | 0.953154 | + | 0.000248 |
| FAM110D | AC093510.2 | 0.956505 | + | 0.000199 |
| FAT4 | AC093510.2 | 0.958593 | + | 0.000172 |
| FBXO4 | AC093510.2 | 0.969056 | + | 7.24E-05 |
| FN1 | AC093510.2 | 0.953801 | + | 0.000238 |
| FOXP1 | AC093510.2 | 0.959036 | + | 0.000167 |
| FOXS1 | AC093510.2 | 0.978149 | + | 2.57E-05 |
| FUOM | AC093510.2 | 0.977247 | + | 2.89E-05 |
| GADD45B | AC093510.2 | 0.961889 | + | 0.000134 |
| GALT | AC093510.2 | 0.964791 | + | 0.000106 |
| GINS2 | AC093510.2 | 0.953093 | + | 0.000249 |
| GLB1L | AC093510.2 | 0.965056 | + | 0.000104 |
| GMPPB | AC093510.2 | 0.95379 | + | 0.000238 |

**Table S5.** (Continued)

| **mRNA** | **LncRNA** | **PCC** | **Interaction** | **P-value** |
| --- | --- | --- | --- | --- |
| GSPT1 | AC093510.2 | 0.952367 | + | 0.000261 |
| HNRNPA0 | AC093510.2 | 0.959632 | + | 0.00016 |
| HSPA5 | AC093510.2 | 0.984376 | + | 9.42E-06 |
| HSPG2 | AC093510.2 | 0.965804 | + | 9.74E-05 |
| HUS1B | AC093510.2 | -0.96823 | - | 7.83E-05 |
| IER3IP1 | AC093510.2 | 0.970434 | + | 6.32E-05 |
| ING3 | AC093510.2 | 0.983798 | + | 1.05E-05 |
| IQGAP2 | AC093510.2 | 0.976495 | + | 3.19E-05 |
| ISPD | AC093510.2 | 0.961422 | + | 0.000139 |
| IST1 | AC093510.2 | -0.95359 | - | 0.000241 |
| KCNJ4 | AC093510.2 | -0.96207 | - | 0.000133 |
| KIF9 | AC093510.2 | 0.983377 | + | 1.13E-05 |
| LOXL4 | AC093510.2 | 0.980288 | + | 1.89E-05 |
| MAP2K6 | AC093510.2 | 0.986038 | + | 6.73E-06 |
| ME2 | AC093510.2 | 0.966067 | + | 9.52E-05 |
| MED17 | AC093510.2 | 0.978034 | + | 2.61E-05 |
| MED29 | AC093510.2 | 0.976089 | + | 3.36E-05 |
| MEGF9 | AC093510.2 | 0.969933 | + | 6.64E-05 |
| MRE11 | AC093510.2 | 0.952487 | + | 0.000259 |
| MRGPRG | AC093510.2 | -0.96823 | - | 7.83E-05 |
| MRPL55 | AC093510.2 | 0.952173 | + | 0.000264 |
| MTUS1 | AC093510.2 | -0.96824 | - | 7.82E-05 |
| MYEF2 | AC093510.2 | 0.966026 | + | 9.56E-05 |
| NCAPG | AC093510.2 | 0.981722 | + | 1.51E-05 |
| NECTIN2 | AC093510.2 | 0.952787 | + | 0.000254 |
| NF2 | AC093510.2 | 0.955208 | + | 0.000217 |
| NUS1 | AC093510.2 | 0.990998 | + | 1.81E-06 |

**Table S5.** (Continued)

| **mRNA** | **LncRNA** | **PCC** | **Interaction** | **P-value** |
| --- | --- | --- | --- | --- |
| NUTF2 | AC093510.2 | 0.956677 | + | 0.000197 |
| NXPH2 | AC093510.2 | 0.981504 | + | 1.56E-05 |
| OLA1 | AC093510.2 | 0.963284 | + | 0.00012 |
| OPN1SW | AC093510.2 | 0.976859 | + | 3.04E-05 |
| OXCT1 | AC093510.2 | 0.954262 | + | 0.000231 |
| P3H2 | AC093510.2 | 0.960727 | + | 0.000147 |
| PDAP1 | AC093510.2 | 0.963786 | + | 0.000116 |
| PDHA1 | AC093510.2 | 0.961524 | + | 0.000138 |
| PDIA3 | AC093510.2 | 0.964065 | + | 0.000113 |
| PF4 | AC093510.2 | 0.957254 | + | 0.000189 |
| PLA2G12B | AC093510.2 | -0.96361 | - | 0.000117 |
| POLA2 | AC093510.2 | 0.95582 | + | 0.000209 |
| POLE2 | AC093510.2 | 0.951912 | + | 0.000268 |
| POLK | AC093510.2 | -0.95277 | - | 0.000254 |
| PSPC1 | AC093510.2 | 0.986928 | + | 5.53E-06 |
| PXMP2 | AC093510.2 | 0.96726 | + | 8.56E-05 |
| PYCARD | AC093510.2 | 0.958184 | + | 0.000177 |
| REEP2 | AC093510.2 | 0.955012 | + | 0.00022 |
| RIN2 | AC093510.2 | 0.97537 | + | 3.67E-05 |
| RNASEH2B | AC093510.2 | 0.972285 | + | 5.21E-05 |
| RRM1 | AC093510.2 | 0.960804 | + | 0.000146 |
| SAAL1 | AC093510.2 | 0.971567 | + | 5.62E-05 |
| SAT1 | AC093510.2 | 0.988297 | + | 3.97E-06 |
| SDHAF4 | AC093510.2 | 0.951387 | + | 0.000277 |
| SELENOS | AC093510.2 | 0.968573 | + | 7.58E-05 |
| SERINC5 | AC093510.2 | 0.984978 | + | 8.38E-06 |
| SFT2D3 | AC093510.2 | 0.96967 | + | 6.82E-05 |

**Table S5.** (Continued)

| **mRNA** | **LncRNA** | **PCC** | **Interaction** | **P-value** |
| --- | --- | --- | --- | --- |
| SKP1 | AC093510.2 | 0.967987 | + | 8.01E-05 |
| SLC45A4 | AC093510.2 | 0.970073 | + | 6.55E-05 |
| SLC7A5 | AC093510.2 | -0.9505 | - | 0.000292 |
| SNRPE | AC093510.2 | 0.984658 | + | 8.92E-06 |
| SPATA24 | AC093510.2 | 0.972673 | + | 5.00E-05 |
| SPATC1 | AC093510.2 | -0.97792 | - | 2.65E-05 |
| SRI | AC093510.2 | 0.96 | + | 0.000155 |
| SRPK1 | AC093510.2 | 0.981264 | + | 1.62E-05 |
| STAT3 | AC093510.2 | -0.95644 | - | 0.0002 |
| SUB1 | AC093510.2 | 0.960715 | + | 0.000147 |
| TEK | AC093510.2 | 0.963178 | + | 0.000121 |
| THOP1 | AC093510.2 | 0.959179 | + | 0.000165 |
| TMED2 | AC093510.2 | 0.990789 | + | 1.94E-06 |
| TMEM231 | AC093510.2 | 0.970044 | + | 6.57E-05 |
| TMEM237 | AC093510.2 | 0.967532 | + | 8.35E-05 |
| TOX3 | AC093510.2 | -0.97612 | - | 3.34E-05 |
| TRA2A | AC093510.2 | 0.979822 | + | 2.02E-05 |
| TTC23L | AC093510.2 | 0.952914 | + | 0.000252 |
| TTC39C | AC093510.2 | -0.96951 | - | 6.92E-05 |
| TXNDC5 | AC093510.2 | 0.959572 | + | 0.00016 |
| TYMS | AC093510.2 | 0.975671 | + | 3.53E-05 |
| UBE2N | AC093510.2 | 0.954926 | + | 0.000221 |
| UQCR10 | AC093510.2 | 0.981968 | + | 1.45E-05 |
| VAPA | AC093510.2 | 0.95376 | + | 0.000239 |
| VBP1 | AC093510.2 | 0.950351 | + | 0.000295 |
| ZDHHC5 | AC093510.2 | -0.9509 | - | 0.000285 |
| ZNF485 | AC093510.2 | -0.95976 | - | 0.000158 |

**Table S5.** (Continued)

| **mRNA** | **LncRNA** | **PCC** | **Interaction** | **P-value** |
| --- | --- | --- | --- | --- |
| ZNF641 | AC093510.2 | 0.975764 | + | 3.49E-05 |
| ZNF670-ZNF695 | AC093510.2 | 0.962978 | + | 0.000123 |
